# Environmental Preferences and Functional Variations of Methanotrophs in Northeast Qinghai‐Tibet Plateau Wetlands

**DOI:** 10.1111/1758-2229.70352

**Published:** 2026-04-30

**Authors:** Kun He, Jiacheng Zhao, Jianbin Pan, Qi Zhang, Sizhong Yang, Huyuan Feng

**Affiliations:** ^1^ Ministry of Education Key Laboratory of Cell Activities and Stress Adaptations School of Life Sciences, Lanzhou University Lanzhou China; ^2^ Cyrosphere Research Station on the Qinghai‐Tibet Plateau, State Key Laboratory of Cryospheric Science, Northwest Institute of Eco‐Environment and Resources, Chinese Academy of Sciences Lanzhou China; ^3^ Center for Grassland Microbiome, Lanzhou University Lanzhou China; ^4^ Center for Excellence in Archaelogical Science and Cultural Heritage, Lanzhou University Lanzhou China

**Keywords:** co‐occurrence network, environmental preference, *pmoA* gene, type I/II methanotrophs, wetlands

## Abstract

Wetlands are important sinks of methane (CH_4_), with CH_4_ oxidation primarily driven by type I (*Gammaproteobacteria*) and type II (*Alphaproteobacteria*) methanotrophs. However, research on the environmental preferences of these two groups in wetlands remains limited. Here, we collected 128 samples, examined soil properties, methanotrophic abundance and community structure and potential CH_4_ oxidation rates (PMORs), across both horizontal (four regions) and vertical (four soil depths) dimensions in the northeastern Qinghai‐Tibet Plateau. We found that the relative abundance of type I and type II methanotrophs, methanotrophic community structure and PMORs were primarily regulated by soil ion concentration (pH and electrical conductivity). Type II methanotrophs prefer environments with low soil ion concentration and high nutrient levels (e.g., soil total organic carbon and total nitrogen), while type I methanotrophs show the opposite trend. Additionally, we found that type I and type II methanotrophs play different roles in wetlands. Type II methanotrophs, especially *Methylocystis*, showed higher PMORs, whereas type I methanotrophs (especially *Methylobacter*, *Methylomonas* and type Id) was crucial for maintaining methanotrophic microbial community stability as keystones. Overall, this study illustrates the disparities in the ecological roles of methanotrophs, thereby enhancing our understanding of microbially mediated carbon cycling in wetland ecosystems.

## Graphical abstract

Significant environmental preference between type I and type II methanotrophs is regulated by soil ion concentration (pH and electrical conductivity) in wetlands. Type II methanotrophs (*Methylocystis*, etc.) contributes more to soil methane oxidation than type I methanotrophs. Type I methanotrophs plays a key connecting role in the MOB community structure.

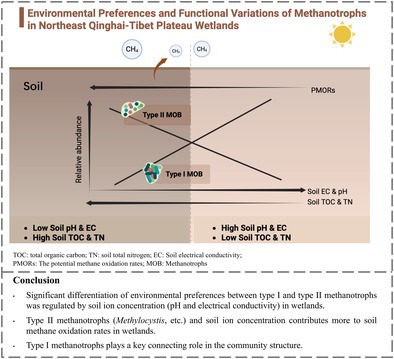

## 1 Introduction

Since the industrial revolution, atmospheric methane (CH_4_) concentrations have risen by approximately 150% (Etminan et al. 2016; Saunois et al. 2020). CH_4_ is a potent greenhouse gas, with a global warming potential 72 times higher than that of carbon dioxide over a 20‐year period (Saunois et al. 2020). Wetlands, though covering only 2%–6% of the Earth's land surface, are critical carbon reservoirs, storing an estimated 1.5 × 10^15^ kg of soil carbon (Kayranli et al. 2010). They act as both sinks and sources of greenhouse gases, regulating atmospheric methane and carbon dioxide levels, and provide essential ecosystem services that contribute to global climate regulation and the achievement of the United Nations Sustainable Development Goals (SDGs) (Mao et al. 2021). Biological CH_4_ flux is the balance output of CH_4_ production and oxidation. Among, the CH_4_ oxidising bacteria (MOB) are widely regarded as the primary contributors to mitigating CH_4_ emissions under various conditions (Yun et al. 2015). It is estimated that 80% of CH_4_ produced by methanogens in anaerobic compartments are consumed by methanotrophs in the oxic‐anoxic interface before escaping to the atmosphere (Conrad and Rothfuss 1991; Frenzel et al. 1992).

The abundance and composition of methanotrophs are key determinants of soil CH_4_ oxidation capacity. In wetlands, two major groups—type I (γ‐proteobacteria) and type II (α‐proteobacteria)—commonly coexist (Ho et al. 2013). These groups show contrasting environmental preferences: type I methanotrophs tend to dominate in environments with high CH_4_ concentrations, such as the Zoige wetland (Yun et al. 2021), whereas type II methanotrophs are predominant in acidic soils rich in organic carbon and total nitrogen, as observed in the Riganqiao peatland (Deng et al. 2013). More broadly, methanotrophic community structure is shaped by multiple environmental factors, including soil electrical conductivity, dissolved organic carbon, pH, total carbon and total nitrogen (Yun et al. 2015; Zhang et al. 2019; Reddy et al. 2020), as well as vegetation type, oxygen availability, nutrient accessibility, and soil moisture have also been confirmed to play important roles in the selection and distribution of methanotrophic species (Singleton et al. 2018; Fang et al. 2022; Roldán et al. 2022). These patterns highlight the diverse ecological niches of MOB. However, systematic investigations into the ecological functions and adaptive differences between type I and type II methanotrophs under varying environmental conditions remain limited. Building upon existing knowledge, further exploration of the CH_4_ oxidation capacities of these two groups under spatially heterogeneous environments is crucial for advancing our understanding of the relationship between biodiversity and ecosystem functioning.

MOB communities are highly complex, consisting of numerous interacting species. Because isolating pure or co‐cultures of methanotrophs remains challenging, correlation‐based network analysis has become a key approach for inferring microbial interactions (Zhang et al. 2020). Although co‐occurrence does not necessarily imply coexistence, network patterns can reveal potential relationships ranging from taxon pairs to multi‐taxon assemblies across ecosystems (Eiler et al. 2012; Tavella and Cagnolo 2019). Such co‐occurrence patterns are often indicative of ecological interactions among microorganisms (Ma et al. 2016). In microbial ecology, ‘keystone taxa’ are often identified based on network topological properties rather than experimental removal approaches (Banerjee et al. 2018). Within Qinghai‐Tibet Plateau wetlands, keystone methanotrophic taxa such as *Methylobacter*, upland soil cluster α (USCα) and rice paddy cluster 1 (RPC‐1) frequently engage in cooperative interactions despite their low abundance, thereby exerting disproportionately large effects on ecosystem functioning (Zhang et al. 2019). However, the interaction patterns between type I and type II MOB, and their potential roles as keystone taxa in stabilising wetland communities, remain poorly understood. Clarifying these roles is critical for advancing our understanding of the ecological processes that sustain wetland ecosystem stability.

The Qinghai‐Tibet Plateau (QTP), often referred to as the ‘Third Pole,’ plays a crucial role in regional and global climate regulation and ecological processes. It is recognised as a significant CH_4_ emission hotspot, with annual emissions estimated at 0.56–1.00 Tg (Ding and Cai 2007). However, its carbon sink capacity has likely been substantially underestimated (Jia et al. 2022). Therefore, systematic studies on the spatial distribution and ecological functions of type I and type II methanotrophs are essential to deepen our understanding of CH_4_ uptake in QTP wetlands and their role in mitigating the greenhouse effect. We collected 128 soil samples from four different wetland ecosystems on the northeastern QTP, which vary in habitat characteristics and soil properties. We analysed soil properties and assessed the abundance and community composition of MOB using quantitative PCR (qPCR) and Illumina MiSeq sequencing. Specifically, we address the following three major questions: (i) Whether and how type I and type II MOB exhibit environmental preference differences in wetlands; (ii) Whether and how type II and type I MOB differ in potential CH_4_ oxidation functionality; and (iii) What are the interactions among MOB microorganisms and keystones in wetlands. In this study, we investigate the ecological roles of coexisting MOB taxa across both horizontal (among sites) and vertical (across soil depths) dimensions within QTP wetland environments. Whereas previous studies have largely focused on a single spatial scale, our research may provide a more comprehensive perspective on microbial‐mediated carbon cycling and help generate new insights into the functions of distinct methanotrophic groups in CH_4_ oxidation.

## 2 Methods

### 2.1 Study Site Description and Sampling

Soil samples were collected in July–August 2020 from wetlands on the northeastern Qinghai–Tibet Plateau, as described in our previous study (He et al. 2025). The four sites included Maqu (MQ) and Luqu (LQ) peatlands in the eastern plateau, and Zhangye (ZY) and Sugan Lake (SGH) desert wetlands in the arid northern region characterised by low precipitation and a dry climate (Zhou et al. 2019) (Table S1; Figure S1).

At each site, eight 2 × 2 m plots were randomly established (≥ 50 m apart). Within each plot, soil was collected from five points (four corners and the centre) after removing aboveground vegetation and homogenised to form one composite sample. Samples were taken at four depths (0–5, 5–10, 10–20 and 20–40 cm). For each depth, four independent composite samples were obtained as biological replicates, with each replicate derived from a distinct set of five non‐overlapping sampling points. In total, 128 soil samples were collected (4 regions × 8 plots × 4 depths). Fresh samples were kept at 4°C in a portable refrigerator during transport. Upon arrival at the laboratory, the samples were divided into two portions: one portion was air‐dried at approximately 25°C and sieved through a 2 mm mesh for physicochemical analyses; the other portion was stored at −20°C for DNA extraction and incubation experiments. A pre‐incubation test was initiated immediately, followed by the formal incubation experiment.

### 2.2 Soil Properties Analysis

Soil physicochemical properties were determined as follows. For pH, 10 g of air‐dried soil was mixed with 50 mL of 1 mol L^−1^ KCl solution, and the pH was measured using a pH metre (Sartorius PB‐10; Sartorius AG, Göttingen, Germany). Electrical conductivity (EC) was measured in a 0.01 mol L^−1^ KCl suspension (1:5 w/v) using a conductivity metre (CT‐3031). Soil moisture content (SWC) was determined by drying fresh soil to a constant weight at 105°C. Total organic carbon (TOC) and total nitrogen (TN) were measured using an Elementar CHNS analyser (Elementar Analysensysteme GmbH, Langenselbold, Germany). Available nitrogen (NO_3_^−^ and NH_4_^+^) was extracted with 2 M KCl (1:5 w/v) and analysed using a FIAstar 5000 Analyser (FOSS, Hillerød, Denmark). Total phosphorus (TP) was determined after digestion with sulfuric acid at 375°C. The soil properties are summarised in Table S2.

Sample preparation and measurement of soil properties followed the same procedures as in our previous research (He et al. 2025). In contrast to our previous study, where soil samples from different depths within a region were pooled for analysis, the present study analysed depth‐specific samples separately in order to better capture vertical heterogeneity in soil properties and microbial communities.

### 2.3 Potential Methane Oxidation Rates (PMORs)

Before incubation, the soil was preincubated at 60% water holding capacity (conducive to the incubation of methanotrophic bacteria) at 25°C in darkness (Yun et al. 2021) for 1 day under ambient air conditions. Aliquots (5 g, dry weight) of the preincubated soil were then placed into 120 mL serum vials capped with two‐way valves and injected CH_4_ into the gas headspace of the vials to yield final mixing ratios of 2% (Siljanen et al. 2011). For each sample, three replicates were prepared, and one sterilised blank control containing only CH_4_ was included to monitor potential CH_4_ loss. No significant CH_4_ depletion was observed in the blanks, which were treated identically to the incubation bottles except for the absence of soil. CH_4_ concentrations were measured on the first and fifteenth day use a gas chromatograph (Agilent 7890, Santa Clara, CA, USA) equipped with a flame ion detector (FID) using 10 mL gas samples from the bottle. The furnace temperature, FID, and ECD detector temperature were 55°C, 200°C and 300°C, respectively. 99.999% high purity nitrogen was selected as the carrier gas, and the flow rate was 2 mL/min. High purity hydrogen and air were used as the gas with flow rates of 40 and 400 mL/min, respectively. Soil potential methane oxidation rates (PMORs) were calculated according to soil incubation time and gas concentration as the following equation:

$$\text{PMORs}=\frac{\mathrm{dc}}{\mathrm{dt}}\cdot \frac{M_{M}\cdot V_{H}\cdot P_{A}}{R\cdot \mathrm{Ws}}\cdot \frac{T_{\mathrm{ST}}}{T_{\mathrm{ST}}+T.}$$

PMORs is the oxidation rates of CH_4_ in soil samples [ng CH_4_ g^−1^ (dry weight) day^−1^]; dc/dt is the rate of change in headspace CH_4_ in the incubation bottle over time (mmol mol^−1^ day^−1^); MM is the molar mass (g mol^−1^) of CH_4_ (g); VH is the volume of serum bottles headspace (L); PA is the atmospheric pressure (kPa); *R* is the gas constant (m^3^ Pa °K^−1^ mol^−1^); WS is the dry weight of soil sample (g); *T*_ST_ and *T* are the standard temperature (°K) and the incubation temperature (°K), respectively (Yang et al. 2022).

### 2.4 DNA Extraction and qPCR of the *pmoA* Gene

DNA extraction was performed from 0.25 g soil samples with DNeasy Power Soil kit (Qiagen, Germantown, MD, United States) according to the manufacturer's instruction. The key enzyme associated with MOB is the particulate methane monooxygenase (pMMO) (Dalton 2005), and the gene *pmoA* is widely recognised as a functional marker for these organisms (Tavormina et al. 2011; Singleton et al. 2018). The copy numbers of *pmoA* gene were determined by real‐time PCR with primer set A189f (5′‐GGNGACTGGGACTTCTGG)‐mb661r (5′‐CCGGMGCAACGTCYTTACC) (Bourne et al. 2001) and a SYBR Green System (Takara Bio Inc., Shiga Japan) as described previously (Costello and Lidstrom 1999; Kolb et al. 2003). Negative controls, samples and standard series were run in triplicate in 96‐well plates. The assays were performed using a Q5 Real‐Time PCR System (Applied Biosystems, Foster City, CA, USA) and the associated software. The 20 μL reaction mixtures contained 2 μL template DNA, 10 μL SYBR Green, 0.4 μL ROX mixture (2×, Takara Bio Inc., Shiga, Japan), 0.4 μL forward primer (10 μmol), 0.4 μL reverse primer (10 μmol L^−1^) and 6.8 μL nuclease‐free water. Quantitative PCR runs started with an initial denaturation and enzyme activation step at 98°C for 2 min, followed by 40 cycles of 10 s at 98°C, 15 s at 62°C and 60 s at 68°C and 10 min at 72°C. Standard curves were constructed using plasmids harbouring the gene fragment. After the qPCR run, a melting curve were constructed to assess the product specificity and potential primer dimer. The *R* ^2^ and amplification efficiency of the standard curve were 0.99% and 96.2%, respectively.

### 2.5 Illumina Sequencing and Bioinformatic Analysis

We used high‐throughput sequencing to characterise the community compositions of MOB. The soil DNA was extracted and then sent to the Majorbio Biotechnology Company (Shanghai, China) for Illumina MiSeq sequencing. For each DNA sample, the *pmoA* gene was PCR‐amplified in triplicate using specific primers (A189f–mb661r) with unique barcodes. The resulting PCR products were then combined and purified prior to library construction. The PCR conditions were: initial denaturation at 94°C for 2 min, followed by 30 cycles of 94°C for 1 min, 60°C for 1 min and 72°C for 1 min, with a final elongation at 72°C for 10 min. Purified PCR products from each sample were quantified, pooled in an equimolar ratio, and used to construct Illumina libraries with the MiSeq Reagent Kit v3 (Illumina, USA). Sequencing was conducted in paired‐end or single‐direction format by Majorbio Company (Shanghai, China) on an Illumina MiSeq PE300 platform. However, due to unsuccessful library preparation of certain DNA samples, the final available sample count is 104 (MQ: 32 samples, LQ: 32 samples, ZY: 27 samples, SGH: 13 samples).

A total of 2,071,622 sequences were obtained from 104 soil samples, corresponding to an average of approximately 19,919 paired‐end sequences per sample. QIIME 2 (2022.8) was used to analyse the sequencing data. Specifically, paired‐end reads were trimmed to a minimum *Q*‐score of 20. The *pmoA* gene sequences were processed to generate amplicon sequence variants (ASVs) by DADA2 (Callahan et al. 2016). This process involved noise filtration, error correction of ambiguous sequences, and the removal of chimeras, singletons and redundant sequences. As a result, representative Amplicon Sequence Variants (ASVs) and a corresponding feature table were obtained. Insertions and deletions caused the frameshifts of the *pmoA* gene sequences were corrected using FrameBot (Wang et al. 2013) from FunGene database (http://fungene.cme.msu.edu) (Cole et al. 2013). The resulting ASVs were taxonomically assigned according to the database from (https://doi.org/10.5880/GFZ.5.3.2016.001) (Yang et al. 2016). To further proofread the taxonomical assignments, the representative sequences of dominant ASVs were compared with the GenBank database using BLAST (https://blast.ncbi.nlm.nih.gov). When presenting results at the genus level, all *pmoA* sequences were classified based on the reference database. Sequences annotated as ‘uncultured’ and those that could not be confidently assigned to any known genus were grouped together and labelled as ‘unclassified’ to maintain consistency in taxonomic resolution. The phylogenetic subtype (e.g., Type Ia, Ib, or Id) was retained when identifiable. The ASVs that contained less than 10 reads were removed from the datasets, and all samples were rarified to 1252 sequences based on the lowest sequencing depth. After processing, the total number of representative ASVs across all samples was 1338. The sequencing depth of amplicon sequencing was estimated using rarefaction analyses. All raw sequencing data of *pmoA* were submitted to the Sequence Read Archive of NCBI under the accession numbers PRJNA1082256 (https://www.ncbi.nlm.nih.gov/bioproject/PRJNA1082256/).

### 2.6 Statistical Analysis

All statistical analyses were performed using R4.2.1 (https://www.R‐project.org/). The data derived from qPCR (i.e., gene copy numbers) were log10 transformed and used in the following analyses. Based on the rarified ASV tables of MOB group, we calculated the ASV richness and the relative abundance of relative abundances of taxonomic groups (i.e., ASV, genus or family) for each sample. To investigate both horizontal and vertical variations in soil properties and MOB communities, we applied one‐way analysis of variance (ANOVA) followed by Tukey's post hoc test to assess differences in soil physicochemical parameters, qPCR results, and ASV α‐diversity among sites and across soil depths within sites.

Regional differences in MOB communities among sites were assessed using constrained analysis of principal coordinates (CAP) with PERMANOVA (Jiao et al. 2016), based on Bray–Curtis distances (‘vegan’ package, R) (Dixon 2003). To identify environmental drivers of ASV variation, CAP with stepwise forward selection and permutation ANOVA (999 permutations, ordistep) was applied, and variance inflation factors (VIF) were examined to avoid multicollinearity. Differences in community composition across soil depths within sites were tested using redundancy analysis (RDA) with PERMANOVA (adonis, 999 permutations). Only soil variables with significant conditional effects (*p* < 0.05, anova.cca) were retained. Soil properties were standardised prior to analysis.

The R package ‘pheatmap’ (https://github.com/raivokolde/pheatmap) was used to explore relationships between soil physicochemical properties and MOB genera, as well as between subnetwork topology and soil properties or MOB communities.

Random forest analysis (RFA) was performed using the ‘randomForest’ package in R (Jin et al. 2020) to predict the factors to PMORs and the relative abundance of MOB.

Regression analyses were conducted to assess relationships among soil properties, relative abundance of MOB, and PMORs.

Network structures were calculated in R using the ‘picante’ package (Kembel et al. 2010) and visualised in Gephi 0.10.1. Co‐occurrence events were defined as Spearman's correlations with *R* > 0.6 and *p* < 0.01. Keystones were classified into four categories—peripherals, connectors, module hubs, and network hubs—based on Zi‐Pi thresholds derived from network topology using the ‘igraph’ package (Olesen et al. 2007; Berry and Widder 2014), with module hubs, connectors, and network hubs considered keystone taxa. Network robustness was assessed by randomly removing nodes and monitoring the decline in natural connectivity, a measure of alternative path redundancy and network stability (Wu Jun et al. 2010). Subnetworks were constructed for each sample using the ‘igraph’ package in R (Berry and Widder 2014). A comprehensive index was established to reflect the complexity of microbial network, and was calculated by averaging the standardised scores of the topological properties including the betweenness centralization, average degree, average neighbourhood, number of nodes, and number of edges, graph density, connectance, global clustering coefficient, degree centralization and average degree centralization (Zhang et al. 2024).

Partial least squares path modelling (PLS‐PM) was used to evaluate potential causal relationships between biotic and abiotic variables and PMORs (Barber et al. 2014; Wagg et al. 2014; Fan et al. 2019). We first grouped the observed soil properties into two latent variables: ‘soil nutrients,’ representing TOC, TN, TP, NH_4_^+^, and NO_3_^−^; and ‘soil ions,’ including pH and EC. The ‘community’ latent variable encompassed MOB richness and the first CAP axis (CAP1) of MOB community composition. Path coefficients were tested against zero using bootstrapping with 1000 resamples, providing estimates of the precision, direction, and strength of direct linear relationships between variables. Indirect effects were calculated as the product of all path coefficients linking a predictor to a response variable, excluding the direct effect. All PLS‐PM analyses were performed in R using the ‘plspm’ package (Monecke and Leisch 2012). The model reliability was evaluated using the Goodness of Fit (GoF) statistic (Monecke and Leisch 2012).

## 3 Results

### 3.1 Diversity and Composition of Soil MOB

**FIGURE 1:**
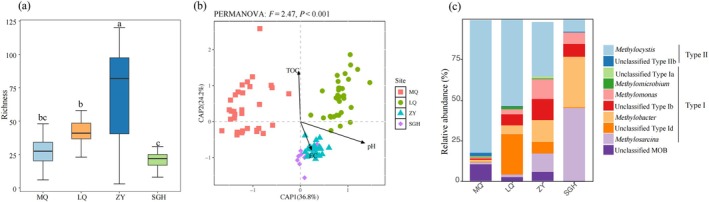
CH_4_ oxidising bacteria (MOB) diversity and composition from *pmoA* gene amplicon data. ASV richness of MOB in the four sites, different letters indicate significant differences at *p* < 0.05 according to Tukey's honest significant difference (HSD) test (a). Constrained analysis of principal coordinates (CAP) showing regional differences in MOB communities among sites. Soil properties were selected using stepwise forward selection with permutation ANOVA (999 permutations). Only selected variables are displayed (b). The relative abundances of top 10 MOB genera in the four sites are shown (c). Sequences that could not be confidently assigned to any known genus, including those annotated as ‘uncultured,’ are grouped as ‘Unclassified.’ Phylogenetic subtypes (Type Ia, Ib, Id) are indicated where applicable. EC, electrical conductivity; LQ, Luqu; MQ, Maqu; SGH, Sugan lake; TOC, total organic carbon; ZY, Zhangye.

Detailed abundance information of all representative ASVs across samples is available in Supporting Information. The alpha diversity of soil MOB communities in the four regions was evaluated in terms of ASV richness, and no significant differences in ASV richness were observed among different depths within the same site (Figure 1a; Table S3). The ASV richness in ZY respectively were strikingly higher than other regions. PERMANOVA revealed that methanotrophic community structure differed significantly among sites (*p* < 0.001; Figure 1b). CAP analysis, combined with forward selection, identified soil pH, TOC, and EC as significant explanatory variables for community composition, with soil pH explaining the largest proportion of variation (Table S4). At the within‐site scale, the MOB community structure differed significantly between MQ and LQ (PERMANOVA, *p* < 0.05), and its variation could be well predicted by soil SWC and TOC (anova.cca by = ‘terms’, *p* < 0.05) (Figure S2, Table S5).

Taxonomic annotations of all ASVs offer limited information, with 94.1% of total ASVs taxonomically assigned only up to the genus level. The MOB genera belonged to two classes (type I and type II MOB) mainly according to their phylogenetic classification. The composition of the top 10 genera differed among the four regions. Type II MOB (*Methylocystis* and type IIb) dominated in MQ (80.4%) and LQ (52.2%), whereas type I MOB, comprising multiple genera, prevailed in ZY (67.7%) and SGH (93.8%) (Figure 1c, Table S6). At MQ, type I MOB increased and type II MOB decreased with depth, while depth‐related differences were generally minor within regions (Table S6).

### 3.2 Difference of *pmoA* Abundance and PMORs in Wetlands

**FIGURE 2:**
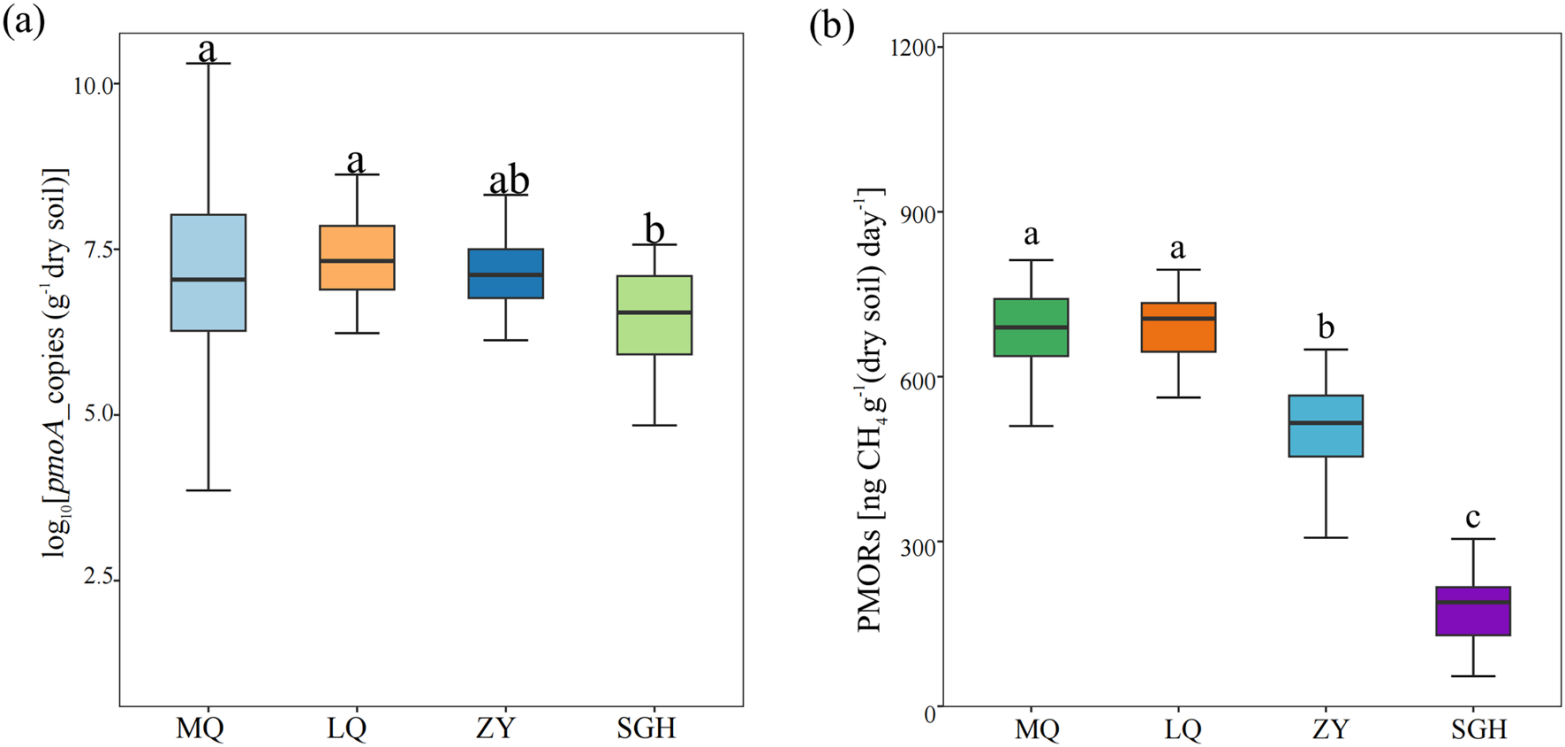
Comparison of *pmoA* gene abundance and the potential methane oxidation rates (PMORs) in wetlands. Comparison of *pmoA* gene abundance of each region (a), the *y* axis is transformed by log10. Comparison of PMORs of each site (b). Different letters are the same as in Figure 1.

In our study, we observed significant differences in *pmoA* gene abundance among the sampled sites, while no variations were found when comparing different depths within the same site (Table S7). The abundance of the *pmoA* gene ranged from (1.24 ± 1.10) × 10^6^ to (2.27 ± 0.49) × 10^7^ copies g^−1^ dry soil (Table S7; Figure 2a). It was significantly higher in the MQ and LQ regions than in SGH, whereas no significant differences were observed between ZY and the other regions.

**FIGURE 3:**
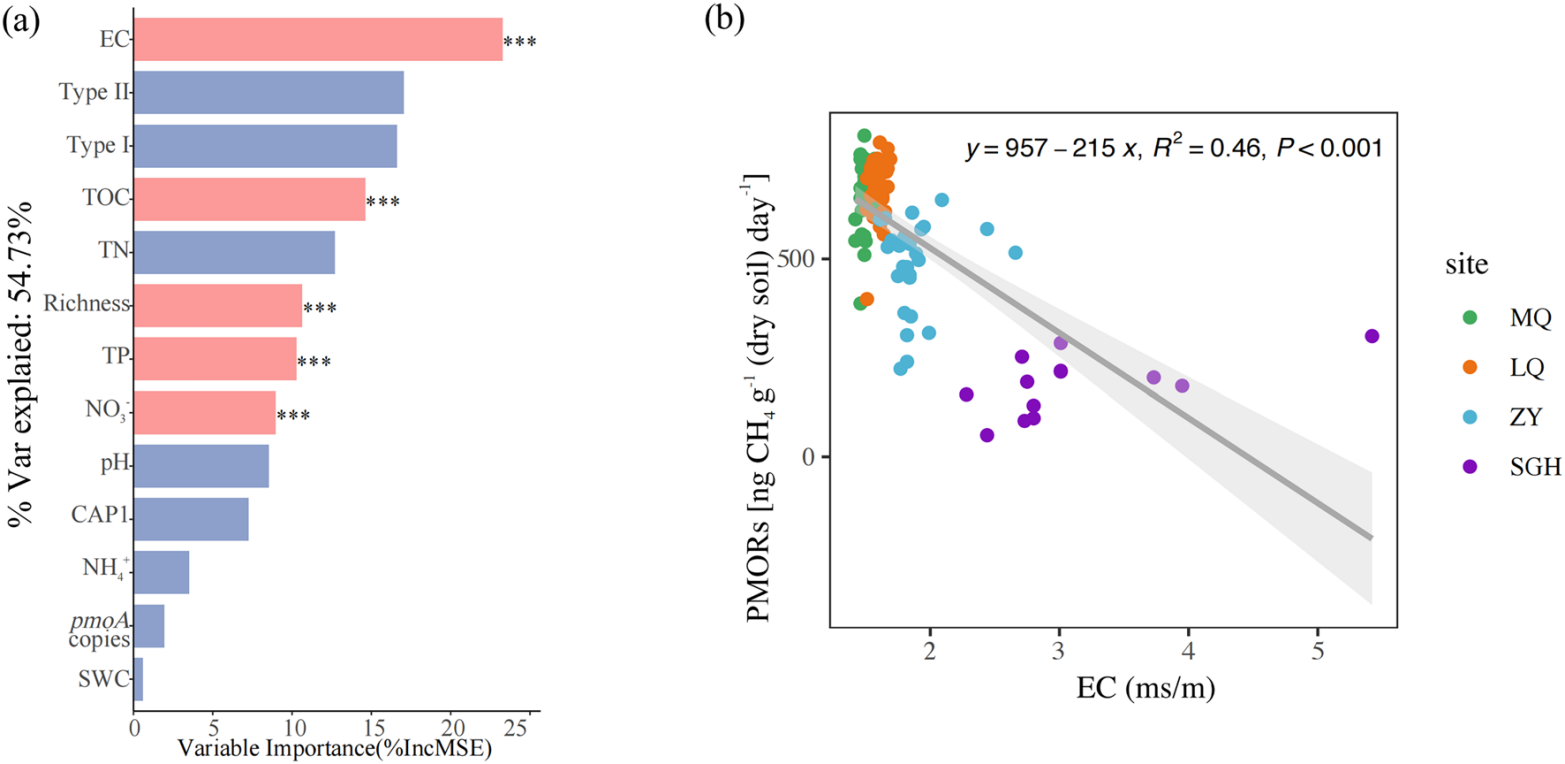
Impact of soil properties on PMORs. Predicted contributions of soil factors to PMORs based on random forest regression analysis (a). ‘% var explained’ meant the goodness of fit of the model. The pink columns indicated the factor that has a significant effect (*p* < 0.05; ANOVA). Regression analysis shows the relationship between soil EC and PMORs across all samples (b).

PMORs ranged from 182.60 ± 20.99 ng g^−1^ dry soil day^−1^ to 683.39 ± 13.99 ng g^−1^ dry soil day^−1^, with significantly higher rates observed in MQ and LQ regions compared to ZY and SGH regions, while no significant differences were observed among different depths within the same site (Table S7). In addition, soil EC emerged as the most significant and influential predictor for PMORs (Figure 3a), showing a negative relationship with PMORs (Figure 3b).

### 3.3 Links Between Soil Properties, Microbial Biodiversity and PMORs

**FIGURE 4:**
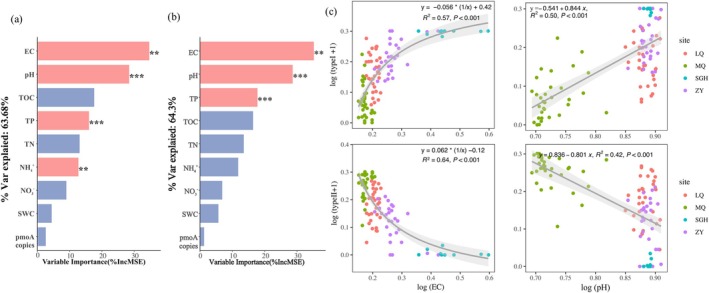
The impact of soil properties on the relative abundance of MOB. The predictions of the soil properties to the relative abundance of type I MOB (a) and type II MOB (b) based on random forest regression analysis. The format is the same as in Figure 3. Regression analysis shows the relationships between soil EC, pH, and the relative abundances of type I/II MOB in all samples, with all data log‐transformed (c).

We employed random forest analysis to investigate environmental factors significantly influencing the relative abundance of type I and type II MOB (Figure 4a,b). The results show that soil EC is the most influential predictor of the relative abundance of both type I and type II MOB, with variable importance exceeding 30%, followed by soil pH as the next most important predictor. The impact of soil EC on the relative abundance of methanotrophic bacteria is far more significant than that of other factors. Regression analysis reveals that the relative abundance of type I and type II MOB respond differently to EC and pH (Figure 4c): type I MOB shows positive correlations with EC and pH, while type II MOB shows negative correlations with EC and pH. Additionally, heatmap analysis based on Wilcoxon distance further confirmed this result (Figure S3): type I MOB is positively correlated with soil ion concentration (soil EC and pH) and negatively correlated with soil nutrients (e.g., TOC, TN), while type II MOB shows the opposite trend.

**FIGURE 5:**
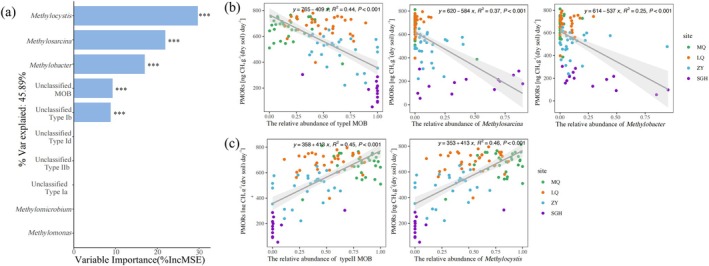
Contribution of type I and type II MOB genera to PMORs. Contributions of different MOB genera to PMORs were assessed using random forest regression analysis, with the format as follows: in Figure 3 (a). Regression analysis shows the correlations between the relative abundances of type I MOB, *Methylosarcina*, and *Methylobacter* (b), and type II MOB and *Methylocystis* (c) with PMORs.

We used random forest analysis to assess the contributions of different MOB genera to PMORs. The analysis revealed that type II MOB, particularly *Methylocystis*, had the strongest predictive effect on PMORs (Figure 5a), followed by *Methylosarcina* and *Methylobacter* from type I MOB. The relative abundance of type I MOB, *Methylosarcina*, and *Methylobacter* exhibits the opposite correlation with PMORs (Figure 5b), while type II MOB and *Methylocystis* show a positive trend (Figure 5c).

**FIGURE 6:**
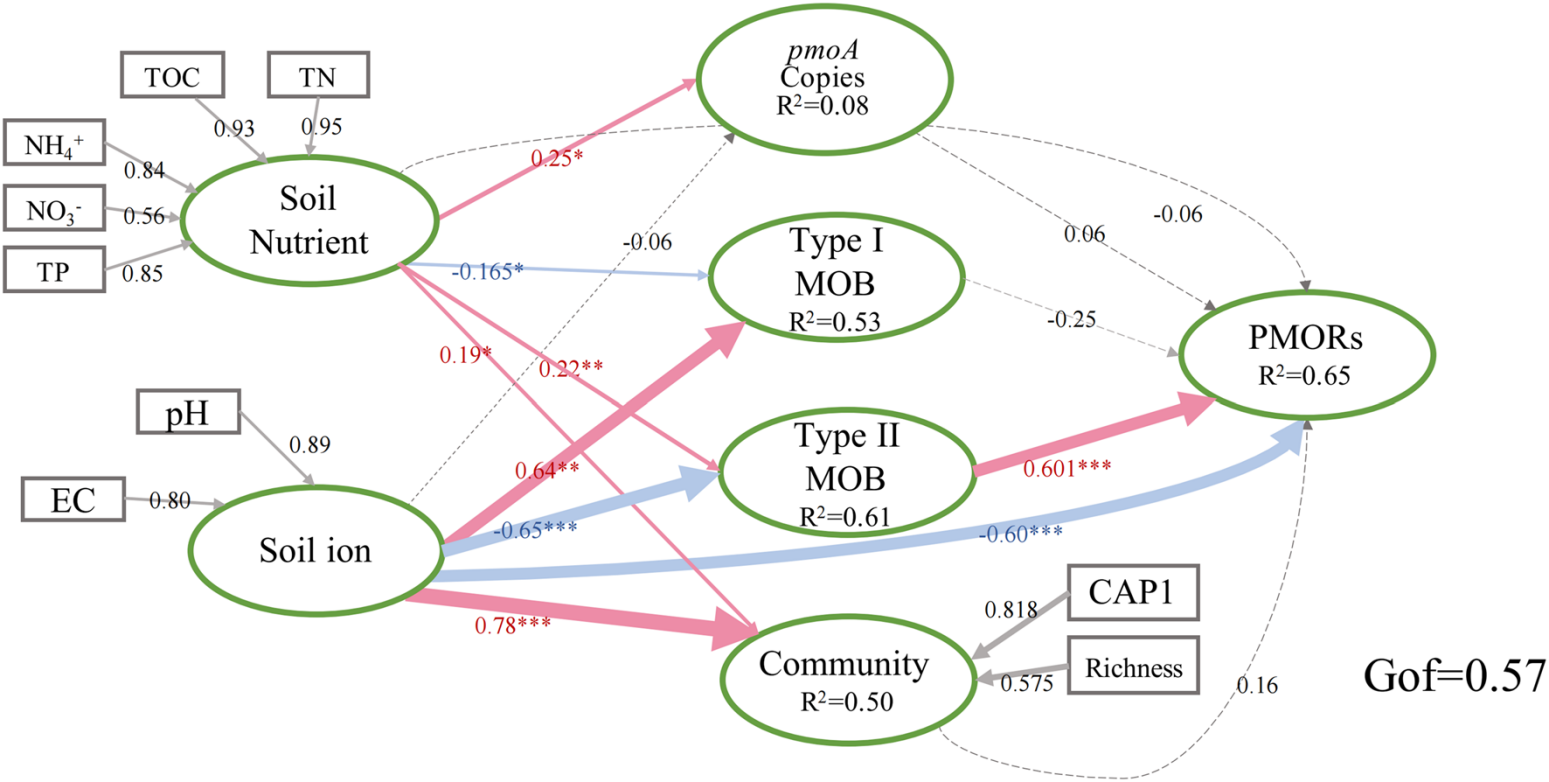
Directed graph of the partial least squares path model (PLS‐PM). Each box represents an observed variables and the ellipse represents observed variables. The model was calculated by the goodness of fit statistic. The weights of arrows indicated the strengths of the causal relationships with red indicating a positive effect and blue a negative effect. Continuous and dashed arrows indicated the significant difference or not. The numbers at the arrows showed the standardised path coefficients. Coefficients of inner model differ significantly from 0 are indicated by *p < 0.05, **p < 0.01, ***p < 0.001. Nutrient: Soil TOC, TN, NH_4_^+^, NO_3_^−^ and TP; type I, the relative abundance of type I; type II, the relative abundance of type II; community: CAP1 and richness; CAP1: The first canonical axis of Canonical Analysis of Principal Coordinates (CAP). *pmoA* copies, *PpmoA* abundance; PMORs, potential methane oxidation rates.

We further explored the direct and indirect effects of soil nutrient, soil ion concentration, *pmoA* abundance, the relative abundance of type I and type II MOB, and the diversity of community on PMORs by PLS‐PM (Figure 6). Generally, PMORs were mainly influenced by soil ion concentration and the relative abundance of type II MOB. Soil nutrient (TOC, TN, NH_4_^+^, NO_3_^−^ and TP) had direct positive effects on *pmoA* abundance (standardised path coefficient = 0.25), the relative abundance of type II MOB (0.22) and the diversity of MOB community (0.19), while it had negative effects on the relative abundance of type I MOB (0.165). The soil ion concentration (pH and EC) had direct positive effects on the relative abundance of type I MOB (0.64) and the MOB community (0.78), negatively affected the relative abundance of type II MOB (0.65) and PMORs (0.60). The goodness of fit of this module is 0.57.

### 3.4 Molecular Ecological Networks and Keystones

**FIGURE 7:**
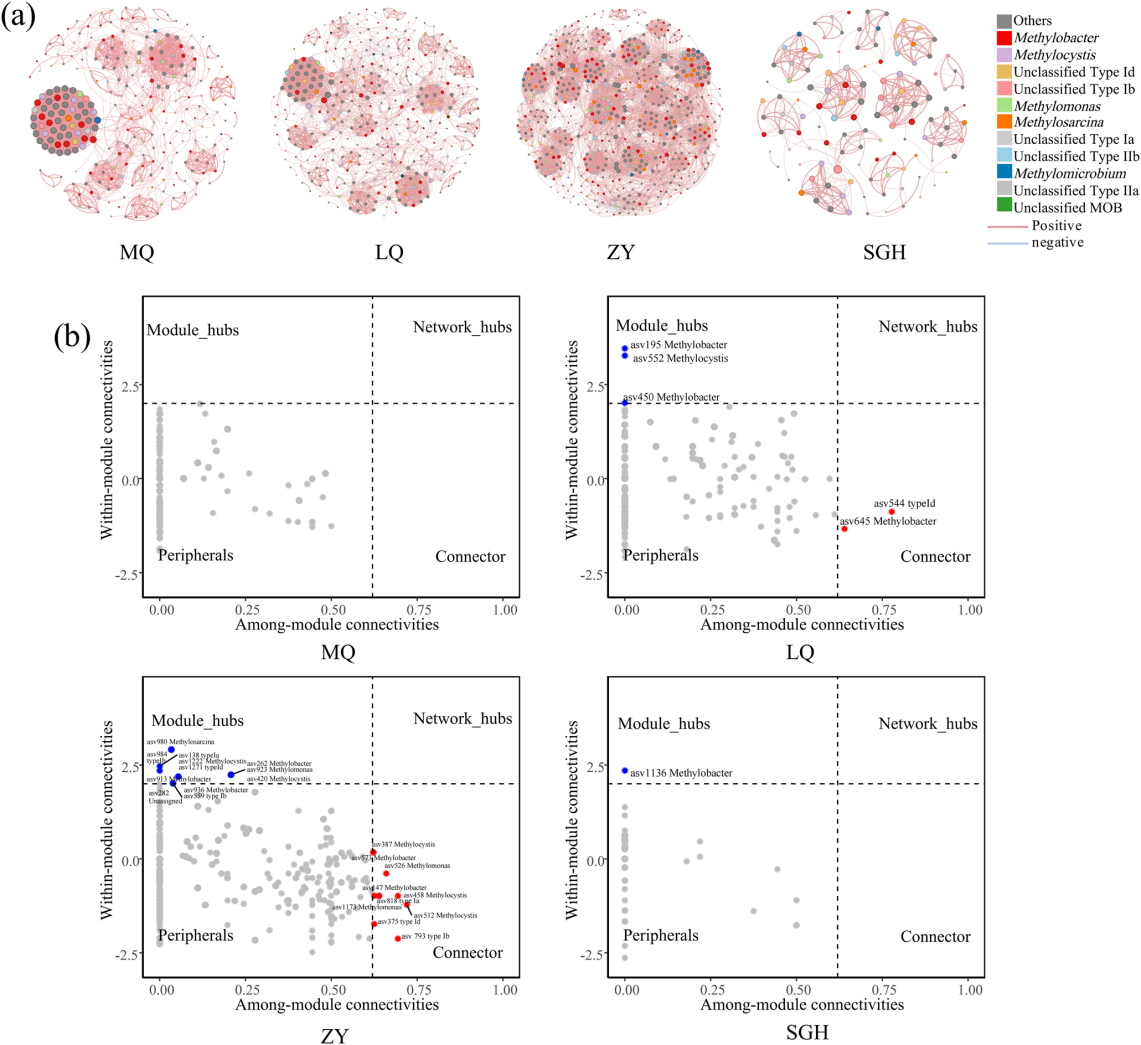
Molecular ecological networks and keystones of MOB communities within four sites. Molecular ecological networks of MOB communities within four sites in MQ, LQ, ZY, SGH (a). Each node represented an ASV. Nodes of different colours are used to differentiate the MOB at the genus level; the size of the nodes showed the number of connections of MOB. The thickness of the edges showed the strength of the correlation among MOB. The pink edges represented positive correlations and blue edges represent negative correlations among ASVs. Zi‐Pi plots showing distribution of ASVs based on their topological roles in MOB networks (b). Threshold values of Zi and Pi for categorising ASVs were 2.5 and 0.62, respectively. LQ, Luqu; MQ, Maqu; SGH, Sugan Lake; ZY, Zhangye.

We analysed microbial networks and key ASVs that connect community nodes and maintain stability across different regions. Molecular ecological networks of MOB were constructed at the genus level for MQ, LQ, ZY and SGH regions (Figure 7a). Table 1 summarises the network parameters, including the number of edges and nodes, positive and negative edges, average degree, natural connectivity, average neighbours, modularity and average geodesic distance. ZY had the most nodes and edges, the smallest geodesic length, and the highest degree, indicating a more complex and interconnected network compared to the others, while SGH had the fewest nodes and edges, suggesting a simpler structure. The distribution patterns of *pmoA* genes in MQ, LQ, and ZY reveal a typical power‐law distribution, suggesting a scale‐free network structure. In contrast, SGH shows a random co‐occurrence pattern, implying random associations between species without a specific structure (Figure S4). The robustness of the network is represented by the average degree and natural connectivity (Figure S5).

**TABLE 1:** Topological properties of molecular ecological networks of methanotrophs community obtained within four sites.

Site	Edges	Nodes	Positive edges	Negative edges	Average degree	Natural connectivity	Average neighbours	Modularity	Average geodesic distance
MQ	2721	286	100%	0%	19.03	39.35	20.49	0.55	2.85
LQ	2330	388	94.88%	0.86%	12.01	22.27	14.07	0.70	3.34
ZY	7575	652	98.20%	0.24%	23.24	31.17	26.90	0.70	2.62
SGH	366	118	100%	0%	6.20	2.92	6.82	0.88	4.07

Abbreviations: LQ, Luqu; MQ, Maqu; SGH, Sugan Lake; ZY, Zhangye.

In addition, we conducted further analysis on the Zi‐Pi relationships among ASVs and found 16 provincial hubs and 11 connectors were identified as keystones in all site (Figure 7b). Among the 27 keystone taxa, 22 were identified as type I MOB, which significantly dominated the keytones in terms of relative abundance (Table S8). Furthermore, analysis of sample‐subnetworks revealed significant differences in network complexity among sites, with the ZY site exhibiting higher complexity than the others, while no significant differences were observed across depths at each site (Figure S6). The relationships between soil properties, MOB richness, the relative abundances of type I and II MOB, *pmoA* gene abundance, and subnetwork topological properties were also examined (Figure S7), we found that subnetwork topology was negatively associated with soil nutrient levels, PMORs and the relative abundance of type II MOB, while showing positive correlations with pH, EC, MOB richness, and the relative abundance of type I MOB.

## 4 Discussion

### 4.1 Environmental Preference Differences of MOB in Wetlands

We observed pronounced geographic differences in the distribution of type I and type II methanotrophic bacteria (MOB) across the four studied regions, with soil pH and total organic carbon (TOC) being significant drivers of community structure (Figure 1; Table S4). These findings are consistent with cultivation experiments showing enrichment of type II MOB under acidic conditions and type I MOB across a broader pH range (Reddy et al. 2020). Similarly, studies in grassland soils reported that pH strongly correlated with MOB composition, with type I MOB more abundant in relatively high‐pH soils (Kolb et al. 2003; Belova et al. 2013; Lima et al. 2014; Kambara et al. 2022). Type II MOB can persist under non‐favourable conditions (e.g., low pH) and are classified as stress‐tolerators (S) or stress‐tolerator–ruderals (S‐R). This strategy enables them to survive under low pH, low nutrient, or other unfavourable conditions, whereas type I MOB, being more ruderals (R) and fast‐growing, are less competitive under such stressful environments (Ho et al. 2013). Type II MOB primarily fix carbon via the serine pathway, whereas type I MOB mainly rely on the ribulose‐monophosphate (RuMP) pathway. The serine pathway has lower energy requirements and is less sensitive to pH under acidic conditions, enabling type II MOB to better sustain growth in acidic soils (Danilova et al. 2013).

Along the depth gradient, pH and EC showed little variation, whereas soil water content (SWC) and total organic carbon (TOC) gradually decreased with increasing depth. This resulted in changes in the methanotrophic bacterial (MOB) community structure at the MQ and LQ sites (Table S2, Figure S2). Correspondingly, the MOB community structure shifted at the MQ and LQ sites (Table S2, Figure S2), with a decrease in the relative abundance of Type II MOB and an increase in Type I MOB. This shift likely reflects differences in carbon conversion efficiency under nutrient‐limited but environmentally stable conditions.

Soil carbon, particularly bioavailable methane‐derived carbon, likely influences niche differentiation of MOB through indirect mechanisms rather than serving as a direct substrate (Zheng et al. 2024). Methanotrophs do not utilise complex organic matter directly; instead, soil carbon may regulate methane availability and shape the physicochemical microenvironment in which MOB operate (Lehmann et al. 2020). The RuMP pathway possessed by Type I methanotrophs converts C1 compounds into biomass more efficiently than the serine pathway used by Type II methanotrophs (Trimmer et al. 2015). Consequently, type I MOB may exhibit higher competitive potential under nutrient‐limited but environmentally stable conditions. In addition, different forms of nitrogen (NH_4_^+^, NO_3_^−^ and urea) can differentially influence Type I and Type II methanotrophs. Both ammonia and urea exerted similar effects on methanotroph communities, leading to an increased proportion of *Methylobacter* and higher overall diversity. In contrast, nitrate had a comparatively weaker impact on community composition (He et al. 2019). Closely related methanotrophic strains exhibit differential responses that allow them to overcome inhibition or toxicity from high nitrogen loads and to assimilate various nitrogen sources, thereby conferring competitive fitness advantages to individual MOB (Hoefman et al. 2014). Collectively, these findings suggest that soil total nitrogen likely shapes MOB community composition through a combination of direct physiological constraints and indirect environmental effects.

Through the synthesis research analysis, we found that soil EC was the factor with the greatest influence on the relative abundance of MOB (Figure 4a,b), Soil pH was the second most significant predictor. However, type I and type II MOB exhibited contrasting trends in their relative abundance in response to variations in ion concentrations (Figures 4c, S3 and 6). This contrasting response further supports the notion that there is a difference in environmental preference between these two groups. The previous research suggested the differences in MOB communities suggest niche separation of MOB across varying salinities, such as dryland salinity, has the potential to alter MOB community and therefore the CH_4_ uptake rates in soils in which disturbance occurs (Bissett et al. 2012). Type II methanotrophs appeared to be unaffected by salt stress at NaCl concentrations up to 0.1 M. Beyond this level, their abundance decreased and remained stable throughout the incubation period, indicating resistance to relatively low salt stress (< 1% NaCl) (Ho et al. 2018). Compared with type II, type I (especially type Ia) methanotrophs might have some specific survival strategies (Shiau et al. 2020), which makes them more resistant to salt stress and dominant in some saline ecosystems. *Methylobacter*, together with other members of the type Ia subgroup (including *Methylosarcina*, *Methylomonas* and *Methylomicrobium* like methanotrophs), constituted the predominantly metabolically active methanotrophs in saline environments (Antony et al. 2010). The apparent stimulation of type Ia methanotrophs may be attributed to increased ammonium bioavailability (Ho et al. 2018). In this study, soil pH and EC significantly influenced the microbial community structure and the relative abundance of MOB. A clear correlation between EC and pH was observed in the soils of this study, making it difficult to distinguish the individual effects of these two factors on the microbial communities.

### 4.2 The Determinants of PMORs in Wetlands

PMORs was calculated based on CH_4_ concentrations measured on Day 1 and Day 15. Although pre‐incubation tests indicated an approximately linear decrease in CH_4_ during this period, the relatively long interval between measurements may have masked potential short‐term fluctuations in methane oxidation rates, particularly during the early incubation stage when microbial activity can change rapidly. Therefore, the calculated values represent average oxidation rates over the incubation period rather than instantaneous rates. While this approach is suitable for comparative analysis across a large number of samples, more frequent sampling would provide a more detailed understanding of CH_4_ oxidation dynamics. Future studies should incorporate additional time points to better capture temporal variations in methane oxidation activity. Random forest analysis identified *Methylocystis* (type II MOB) as the primary contributor to CH_4_ oxidation, with a variable importance exceeding 30% (Figures 5a and 6). Moreover, soil PMORs exhibited a significant positive correlation with the relative abundance of type II methanotrophs, but a significant negative correlation with type I methanotrophs (Figure 5). These findings suggest that CH_4_ oxidation strengthens with a higher proportion of type II MOB and weakens with a higher proportion of type I, indicating that type II MOB possess superior CH_4_ oxidation capability in this study. Previous studies have also shown that *Methylocystis* plays a crucial role in CH_4_ oxidation in soils exposed to atmospheric methane (Cai et al. 2016). Some species in genus *Methylocystis* in type II methanotrophs, have been reported to have a relatively high affinity for CH_4_ (Baani and Liesack 2008; Im et al. 2011; Limbri et al. 2014). Under fluctuating conditions of feast and famine, the most abundant and active methanotrophs belong to the type II genus *Methylocystis*, accounting for 91%–65% of the MOB community (Cantera et al. 2023). Further, as a member of the Rhizobiales, *Methylocystis*—along with most type II methanotrophs—possesses nitrogen‐fixing capabilities, whereas only some type I methanotrophs do (Auman et al. 2001). By utilising CH_4_ as an electron donor to drive biological nitrogen fixation (Guo et al. 2022), *Methylocystis* enhances its metabolic flexibility, thereby promoting CH_4_ oxidation efficiency.

Soil ion concentration (EC and pH) is the main factor negatively impacting PMORs (Figure 6). High soil salinity increases osmotic stress, decreases water availability, and limits CH_4_ oxidation (Dalal et al. 2008). Consequently, we propose that the observed trends can be attributed to the enhanced adaptability of type II methanotrophs to nutrient‐rich environments, such as those present in MQ and LQ. In arid ecosystems like desert wetlands (ZY and SGH), the acquisition of organic matter is reduced, which in turn limits CH_4_ oxidation.

A limitation of this study is that in situ gas measurements could not be obtained due to sampling constraints. Future studies should aim to include in situ gas measurements to provide a more comprehensive understanding of the processes under investigation.

### 4.3 Type I MOB May Be the Keystone Taxa in the Community

In natural ecological settings, species do not exist in isolation but form intricate network systems through interactions with other species (Ma et al. 2016). These network structures provide us with deeper insights into the interconnections among microbes and the ecological assembly rules, far beyond the mere understanding of diversity and community composition (Ziegler et al. 2018). In this study on co‐occurrence networks, weaker correlations were filtered out. Correlation analysis revealed predominantly positive relationships among MOB across all habitats (see Table 1), signifying cooperative rather than competitive interactions within these communities.

Methanotrophic networks exhibited power‐law distribution patterns (scale‐free networks) in MQ, LQ and ZY. This means that the power‐law distribution networks have many nodes with few links and a few highly connected nodes that are termed as hubs (Faust et al. 2012). Networks with these characteristics are considered to be robust towards random node removal but sensitive to the removal of hub nodes. In LQ, although type II MOB is the dominant species, type I MOB plays a more critical role in community cooperation, the critical keystone taxa at this site are *Methylobacter* (Table S7).

Soil properties were significantly correlated with subnetwork topological features in each sample (Figure S7), suggesting that MOB diversity is closely linked to methanotrophic interactions. Within the community, type I MOB act as a critical linchpin, maintaining overall coherence and function, whereas type II MOB do not appear to promote interactions among ASVs. Moreover, network connectivity and complexity do not necessarily enhance the community's PMORs. In other studies on wetland systems in the QTP, similar results have also been found, namely that type I methanotrophs are considered keystones that may play an important role in maintaining the stability of wetland community structure from Zi‐Pi space (Zhang et al. 2019). From the perspective of microbial co‐occurrence networks, our results suggest that type I MOB can be identified as keystone taxa. However, this inference is limited by the fact that network correlations do not necessarily represent direct ecological interactions and may be influenced by environmental factors. Future work combining experimental validation and multi‐omics analyses will be essential to confirm the functional roles of these potential keystone taxa.

## 5 Conclusion

This study reveals the environmental preferences of type I and type II methanotrophs and highlights the functional roles of lineage‐specific methanotrophic groups in QTP wetland soils. Our results show that methanotroph community composition is closely linked to potential methane oxidation rates in response to changes in soil properties, providing a theoretical basis for predicting methane fluxes and carbon cycling. Future studies should employ more sensitive approaches, such as in situ gas flux measurements combined with multi‐omics techniques, and further investigate the roles of methanotrophs in key carbon cycling processes, as well as the relationships between methane‐cycling genes, microbial communities and these ecosystem functions.

## Author Contributions

**Kun He:** conceptualization, investigation, writing – original draft, writing – review and editing, methodology, software, data curation, supervision, visualization, formal analysis. **Jiacheng Zhao:** writing – review and editing, conceptualization, methodology, investigation, supervision. **Jianbin Pan:** investigation, funding acquisition, writing – review and editing, visualization. **Qi Zhang:** funding acquisition, investigation, supervision. **Sizhong Yang:** writing – review and editing, software, methodology. **Huyuan Feng:** validation, writing – review and editing, funding acquisition, project administration, resources.

## Funding

This work was supported by the Second Tibetan Plateau Scientific Expedition and Research Program (2019QZKK0301); the National Natural Science Foundation of China (U21A20186, 32371592, 42271155 and 32361133551) and Natural Science Foundation of Gansu Province (23JRRA1034).

## Conflicts of Interest

The authors declare no conflicts of interest.

## Supporting information


**Data S1:** emi470352‐sup‐0001‐TableS1.xlsx.

**Table S1:** The geographic locations and basic informations of all plots. LQ, Luqu; MAP, mean annual precipitation; MAT, mean annual temperature; MQ, Maqu; SGH, Sugan lake; ZY, Zhangye. This table is identical to that published in our previous study (He et al., 2025).
**Table S2:** Comparison of soil characteristics among soil depths within each site and across the four study sites. Letters following the site names (e.g., MQa, MQb, MQc …) denote soil depths from top to bottom. Different letters indicate significant differences at *p* < 0.05 according to Tukey's honest significant difference (HSD) test. EC, electrical conductivity; LQ, Luqu; MQ, Maqu; NH_4_^+^, soil ammonium; NO_3_^−^, soil nitrate; SGH, Sugan lake; SWC, soil water content; TN, total nitrogen; TOC, total oiganic carbon; TP, total phosphorus; ZY, Zhangye.
**Table S3:** MOB alpha diversity index. For abbreviations, see Table S2.
**Table S4:** CAP analysis with forward selection and permutation ANOVA of soil properties influencing MOB community structure. For abbreviations, see Table S2.
**Table S5:** PERMANOVA results for *β*‐diversity of MOB across depths within each site. For abbreviations, see Table S2.
**Table S6:** Relative abundance of MOB community in different site based on class and genus level. For abbreviations, see Table S2. All type I/II: Total relative abundance of all type I/II MOB.
**Table S7:** Comparison of PMORs and *pmoA* gene abundance. For abbreviations, see Table S2.
**Table S8:** The nodes identified as connectors/Module‐hubs of MOB networks in four sites. For abbreviations, see Table S2.
**Figure S1:** Research sites on the edge of northeastern Qinghai‐Tibet Plateau. The data is provided by STRMdem (https://earthexplorer.usgs.gov/). This figure is identical to that published in our previous study (He et al., 2025). For abbreviations, see Table S2.
**Figure S2:** Community composition of MOB across soil depths at each site and their potential drivers. Shown are RDA ordination biplots with only significant explanatory variables (*p* < 0.05, conditional effects). For abbreviations, see Table S2.
**Figure S3:** Heatmap analysis between the environmental factors and the top 10 genus relative abundance based on Wilcoxon Distance. ***p < 0.001; **p < 0.01; *p < 0.05. For abbreviations, see Table S2.
**Figure S4:** Network topological features of degree distribution patterns in four habitats. For abbreviations, see Table S2.
**Figure S5:** The average degree and natural connectivity of microbial network within four sites. For abbreviations, see Table S2.
**Figure S6:** Comparison of microbial subnetwork complexity across soil depths at four sites (a) and among sites (b). In (a), different letters denote soil depths from top to bottom. In (b), different letters indicate significant differences (*p* < 0.05) according to Tukey's honest significant difference (HSD) test. Group differences were evaluated using one‐way ANOVA. For abbreviations, see Table S2.
**Figure S7:** Heatmap analysis between the soil properties, MOB richness, type I/II MOB relative abundance, *pmoA* copies and the subnetwork topological properties based on Wilcoxon Distance. ***p < 0.001; **p < 0.01; *p < 0.05. For abbreviations, see Table S2.

## Data Availability

All raw sequencing data of *pmoA* were submitted to the Sequence Read Archive of NCBI under the accession numbers PRJNA1082256 (https://www.ncbi.nlm.nih.gov/bioproject/PRJNA1082256/). Other data supporting the findings of this study are available from the corresponding author upon reasonable request. Due to privacy or ethical restrictions, these data are not publicly available.
