# Supplementary material for: Environmental Preferences and Functional Variations of Methanotrophs in Northeast Qinghai‐Tibet Plateau Wetlands
Source: Environ Microbiol Rep. 2026 Apr 30;18(3):e70352. doi: 10.1111/1758-2229.70352 (PMC13132679; doi:10.1111/1758-2229.70352)
Supplement: Supplementary file 2 — Table S1: The geographic locations and basic informations of all plots. LQ, Luqu; MAP, mean annual precipitation; MAT, mean annual temperature; MQ, Maqu; SGH, Sugan lake; ZY, Zhangye. This table is identical to that published in our previous study (He et al., 2025). Table S2: Comparison of soil characteristics among soil depths within each site and across the four study sites. Letters following the site names (e.g., MQa, MQb, MQc …) denote soil depths from top to bottom. Different letters indicate significant differences at p < 0.05 according to Tukey's honest significant difference (HSD) test. EC, electrical conductivity; LQ, Luqu; MQ, Maqu; NH4 +, soil ammonium; NO3 −, soil nitrate; SGH, Sugan lake; SWC, soil water content; TN, total nitrogen; TOC, total oiganic carbon; TP, total phosphorus; ZY, Zhangye. Table S3: MOB alpha diversity index. For abbreviations, see Table S2. Table S4: CAP analysis with forward selection and permutation ANOVA of soil properties influencing MOB community structure. For abbreviations, see Table S2. Table S5: PERMANOVA results for β‐diversity of MOB across depths within each site. For abbreviations, see Table S2. Table S6: Relative abundance of MOB community in different site based on class and genus level. For abbreviations, see Table S2. All type I/II: Total relative abundance of all type I/II MOB. Table S7: Comparison of PMORs and pmoA gene abundance. For abbreviations, see Table S2. Table S8: The nodes identified as connectors/Module‐hubs of MOB networks in four sites. For abbreviations, see Table S2. Figure S1: Research sites on the edge of northeastern Qinghai‐Tibet Plateau. The data is provided by STRMdem (https://earthexplorer.usgs.gov/). This figure is identical to that published in our previous study (He et al., 2025). For abbreviations, see Table S2. Figure S2: Community composition of MOB across soil depths at each site and their potential drivers. Shown are RDA ordination biplots with only significant explanatory variables [file EMI4-18-e70352-s001.docx]

**Supplementary materials**

**Environmental Preferences and Functional Potential of Methanotrophs in Northeast Qinghai-Tibet Plateau Wetlands**

Kun He^a^, Jiacheng Zhao^a^, Jianbin Pan^a^, Qi Zhang^a^, Sizhong Yang^c^, Huyuan Feng^a,b,c*^

*﻿^a^ Ministry of Education Key Laboratory of Cell Activities and Stress Adaptations, School of Life Sciences, Lanzhou University, Lanzhou, 730000, China.*

*^b^* *Center for Excellence in Archaelogical Science and Cultural Heritage, Lanzhou University, Lanzhou, 730000, China.*

*^c^* *Center for Grassland Microbiome, Lanzhou University, Lanzhou, 730000, China*

*﻿^d^* *Cyrosphere Research Station on the Qinghai-Tibet Plateau, State Key Laboratory of Cryospheric Science, Northwest Institute of Eco-Environment and Resources, Chinese Academy of Sciences, Donggang West Road 320, Lanzhou, 730000, China.*

*Corresponding author: fenghy@lzu.edu.cn (H. Feng)

**Table S1.** The geographic locations and basic informations of all plots. MQ: Maqu; LQ: Luqu; ZY: Zhangye; SGH: Sugan lake; MAT: mean annual temperature; MAP: mean annual precipitation. This table is identical to that published in our previous study (He et al., 2025).

| plot | Longtitude (E) | Latitude (N) | MAT (℃) | MAP (mm) | Elevation (m) |
| --- | --- | --- | --- | --- | --- |
| MQ1 | 101.6750 | 33.7577 | 1.20 | 717 | 3503 |
| MQ2 | 101.6727 | 33.7579 | 1.17 | 716 | 3503 |
| MQ3 | 101.6764 | 33.7582 | 1.20 | 717 | 3504 |
| MQ4 | 101.6738 | 33.7592 | 1.15 | 716 | 3511 |
| MQ5 | 101.8669 | 33.6637 | 1.21 | 711 | 3500 |
| MQ6 | 101.8657 | 33.6631 | 1.23 | 710 | 3511 |
| MQ7 | 101.8669 | 33.6621 | 1.21 | 711 | 3511 |
| MQ8 | 101.8682 | 33.6627 | 1.21 | 711 | 3511 |
| LQ1 | 102.4083 | 34.2083 | 1.26 | 651 | 3475 |
| LQ2 | 102.4081 | 34.2087 | 1.30 | 651 | 3477 |
| LQ3 | 102.4086 | 34.2088 | 1.28 | 651 | 3489 |
| LQ4 | 102.4094 | 34.2087 | 1.28 | 645 | 3440 |
| LQ5 | 102.3064 | 34.3000 | 1.35 | 645 | 3440 |
| LQ6 | 102.3071 | 34.2998 | 1.35 | 645 | 3440 |
| LQ7 | 102.3071 | 34.2993 | 1.35 | 645 | 3440 |
| LQ8 | 102.3065 | 34.2997 | 1.35 | 645 | 3440 |
| ZY1 | 100.4572 | 38.9691 | 7.05 | 188 | 1461 |
| ZY2 | 100.4572 | 38.9692 | 7.05 | 188 | 1461 |
| ZY3 | 100.4571 | 38.9692 | 7.05 | 188 | 1461 |
| ZY4 | 100.4572 | 38.9693 | 7.05 | 178 | 1461 |
| ZY5 | 100.4039 | 39.0887 | 7.30 | 178 | 1423 |
| ZY6 | 100.4039 | 39.0886 | 7.30 | 178 | 1423 |
| ZY7 | 100.4040 | 39.0887 | 7.30 | 178 | 1423 |
| ZY8 | 100.4041 | 39.0887 | 7.30 | 178 | 1423 |
| SGH1 | 93.9123 | 38.8994 | 1.95 | 46 | 2794 |
| SGH2 | 93.9131 | 38.8993 | 2.17 | 46 | 2809 |
| SGH3 | 93.9137 | 38.8993 | 2.17 | 46 | 2809 |
| SGH4 | 93.9150 | 38.8996 | 2.17 | 46 | 2809 |
| SGH5 | 94.1814 | 39.0548 | 2.17 | 46 | 2809 |
| SGH6 | 94.1822 | 39.0569 | 2.17 | 46 | 2809 |
| SGH7 | 94.1817 | 39.0559 | 2.17 | 46 | 2809 |
| SGH8 | 94.1810 | 39.0540 | 2.17 | 46 | 2809 |

**Table S2.** Comparison of soil characteristics among soil depths within each site and across the four study sites. Letters following the site names (e.g., MQa, MQb, MQc …) denote soil depths from top to bottom. Different letters indicate significant differences at *P* < 0.05 according to Tukey’s honest significant difference (HSD) test. MQ: Maqu; LQ: Luqu; ZY: Zhangye; SGH: Sugan lake; SWC: soil water content; TOC: total oiganic carbon; TN: total nitrogen; TP: total phosphorus; NO_3_^-^: soil nitrate; NH_4_^+^: soil ammonium; EC: electrical conductivity.

|  | MQa | MQb | MQc | MQd |
| --- | --- | --- | --- | --- |
| SWC (%) | 213.142(49.501)a | 135.096(16.248)ab | 71.428(15.378)b | 47.249(8.105)b |
| TOC (mg/g) | 93.458(6.193)a | 67.417(7.318)b | 33.274(5.035)c | 19.163(5.468)c |
| TN (mg/g) | 13.385(1.089)a | 9.024(0.871)b | 4.379(0.620)c | 2.364(0.581)c |
| NH_4_^+^ (mg/kg) | 26.263(4.446)a | 14.918(1.063)b | 7.971(0.452)b | 6.953(0.670)b |
| NO_3_^-^ (mg/kg) | 6.828(3.523)a | 2.538(0.909)a | 2.388(0.377)a | 1.999(0.334)a |
| TP (mg/g) | 1.297(0.071)a | 1.365(0.069)a | 0.943(0.088)b | 0.625(0.070)c |
| pH | 5.095(0.023)a | 5.334(0.187)a | 5.495(0.148)a | 5.498(0.108)a |
| EC (ms/m) | 1.542(0.018)a | 1.496(0.013)ab | 1.475(0.009)b | 1.461(0.008)b |
|  | LQa | LQb | LQc | LQd |
| SWC (%) | 190.178(29.635)a | 142.622(12.733)ab | 104.676(6.947)bc | 64.505(5.071)c |
| TOC (mg/g) | 78.918(5.927)a | 72.890(5.296)ab | 58.498(4.190)b | 37.403(3.280)c |
| TN (mg/g) | 10.889(0.956)a | 10.141(0.807)a | 8.208(0.599)a | 4.303(0.377)b |
| NH_4_^+^ (mg/kg) | 19.282(3.390)a | 8.641(0.873)b | 5.569(0.754)b | 3.616(0.299)b |
| NO_3_^-^ (mgk/g) | 10.784(1.966)a | 8.502(0.914)a | 6.595(0.678)a | 6.811(0.783)a |
| TP (mg/g) | 0.952(0.068)a | 0.787(0.103)ab | 0.672(0.081)ab | 0.528(0.063)b |
| pH | 7.598(0.100)a | 7.588(0.073)a | 7.696(0.070)a | 7.683(0.122)a |
| EC (ms/m) | 1.654(0.011)a | 1.625(0.009)ab | 1.586(0.012)b | 1.541(0.011)c |
|  | ZYa | ZYb | ZYc | ZYd |
| SWC (%) | 60.801(8.908)a | 60.678(9.322)a | 56.146(15.020)a | 39.999(2.547)a |
| TOC (mg/g) | 18.512(0.983)a | 15.300(0.780)ab | 12.941(0.724)b | 12.813(2.403)b |
| TN (mg/g) | 1.557(0.276)a | 1.062(0.081)ab | 0.809(0.108)b | 0.854(0.116)b |
| NH_4_^+^ (mg/kg) | 5.080(0.365)a | 4.808(0.318)a | 4.584(0.280)a | 5.959(0.844)a |
| NO_3_^-^ (mg/kg) | 1.466(0.156)a | 1.473(0.161)a | 1.321(0.119)a | 1.316(0.167)a |
| TP (mg/g) | 0.481(0.030)a | 0.480(0.024)a | 0.528(0.047)a | 0.553(0.062)a |
| pH | 7.691(0.111)a | 7.662(0.046)a | 7.750(0.073)a | 7.744(0.089)a |
| EC (ms/m) | 1.964(0.130)a | 1.787(0.051)a | 1.843(0.025)a | 1.897(0.092)a |
|  | SGHa | SGHb | SGHc | SGHd |
| SWC (%) | 71.369(25.612)a | 95.630(28.042)a | 76.953(21.428)a | 71.909(25.293)a |
| TOC (mg/g) | 28.538(8.182)a | 37.980(5.938)a | 35.435(3.336)a | 38.874(5.304)a |
| TN (mg/g) | 1.974(0.542)a | 2.335(0.897)a | 1.785(0.616)a | 1.949(0.721)a |
| NH_4_^+^ (mg/kg) | 9.975(1.754)a | 7.312(1.618)a | 5.407(0.783)a | 6.235(1.086)a |
| NO_3_^-^ (mg/kg) | 1.845(0.261)a | 1.265(0.097)a | 1.380(0.391)a | 2.328(0.093)a |
| TP (mg/g) | 0.401(0.018)a | 0.376(0.017)ab | 0.357(0.022)ab | 0.292(0.040)b |
| pH | 8.002(0.279)a | 7.703(0.059)a | 7.780(0.010)a | 7.800(0.010)a |
| EC (ms/m) | 3.792(0.568)a | 3.010(0.493)a | 2.847(0.084)a | 2.633(0.097)a |
|  | MQ | LQ | ZY | SGH |
| SWC (%) | 116.729(17.420)a | 125.495(11.511)a | 54.174(5.019)b | 78.381(11.437)ab |
| TOC (mg/g) | 53.328(5.963)ab | 61.927(3.662)a | 14.876(0.821)c | 34.694(3.089)bc |
| TN (mg/g) | 7.288(0.859)a | 8.385(0.572)a | 1.071(0.099)b | 2.008(0.302)b |
| NH_4_^+^ (mg/kg) | 14.026(1.769)a | 9.277(1.382)b | 5.119(0.265)b | 7.443(0.824)b |
| NO_3_^-^ (mg/kg) | 3.438(0.941)b | 3.438(0.941)b | 1.391(0.073)b | 1.715(0.160)b |
| TP (mg/g) | 1.057(0.064)a | 0.735(0.047)b | 0.511(0.022)c | 0.360(0.016)c |
| pH | 5.355(0.069)b | 7.641(0.045)a | 7.714(0.041)a | 7.835(0.085)a |
| EC (ms/m) | 1.494(0.008)c | 1.602(0.009)c | 1.876(0.043)b | 3.126(0.230)a |

**Table S3.** MOB alpha diversity index. For abbreviations, see Table S2.

|  | MQa | MQb | MQc | MQd |
| --- | --- | --- | --- | --- |
| Richness | 29.375(5.060)a | 47.000(6.231)a | 46.875(7.247)a | 32.750(4.122)a |
| Shannon | 1.994(0.227)a | 2.612(0.238)a | 2.629(0.266)a | 2.317(0.252)a |
|  | LQa | LQb | LQc | LQd |
| Richness | 58.375(4.709)a | 55.125(6.770)a | 67.500(7.780)a | 52.875(4.726)a |
| Shannon | 2.899(0.125)a | 2.762(0.110)a | 3.002(0.137)a | 2.962(0.067)a |
|  | ZYa | ZYb | ZYc | ZYd |
| Richness | 102.286(5.295)a | 83.667(19.320)a | 83.143(17.048)a | 82.286(14.089)a |
| Shannon | 3.809(0.072)a | 3.234(0.586)a | 3.213(0.302)a | 3.247(0.412)a |
|  | SGHa | SGHb | SGHc | SGHd |
| Richness | 47.500(11.836)a | 71.333(5.487)a | 46.667(13.371)a | 42.333(11.837)a |
| Shannon | 2.258(0.268)a | 2.276(0.088)a | 2.278(0.507)a | 2.133(0.328)a |
|  | MQ | LQ | ZY | SGH |
| Richness | 28.906(2.488)bc | 43.250(2.184)b | 69.519(7.293)a | 21.000(2.082)c |
| Shannon | 2.281(0.129)b | 2.806(0.057)a | 3.196(0.202)a | 1.863(0.127)b |

**Table S4.** CAP analysis with forward selection and permutation ANOVA of soil properties influencing MOB community structure. For abbreviations, see Table S2.

| Factor | Df | AIC | F | *P*-value | Signif. |
| --- | --- | --- | --- | --- | --- |
| pH | 1 | 718.197 | 1.774 | 0.001 | *** |
| TOC | 1 | 718.851 | 1.315 | 0.001 | *** |
| EC | 1 | 718.465 | 1.343 | 0.01 | ** |

Significance: 0 = ‘***’; 0.001 = ‘**’; 0.01 = ‘*’; 0.05 = ‘.’; 0.1 = ''

Df = degrees of freedom.

Permutations = 999

Overall model significance: Pseudo-F = 2.47, *P* = 0.001.

**Table S5.** PERMANOVA results for *β*-diversity of MOB across depths within each site. For abbreviations, see Table S2.

| Site | df | F-value | *R^2^* | *P*-value |
| --- | --- | --- | --- | --- |
| MQ | 3 | 3.268 | 0.259 | 0.001*** |
| LQ | 3 | 2.102 | 0.183 | 0.006** |
| ZY | 3 | 0.741 | 0.09 | 0.941 |
| SGH | 3 | 0.610 | 0.168 | 0.969 |

**Table S6.** ﻿Relative abundance of MOB community in different site based on class and genus level. For abbreviations, see Table S2. **All type I/II**: Total relative abundance of all type I/II MOB.

| class | genera | MQa | MQb | MQc | MQd |
| --- | --- | --- | --- | --- | --- |
| Type II | *Methylocystis* | 0.944(0.021)a | 0.754(0.073)a | 0.762(0.050)a | 0.815(0.043)a |
|  | Unclassified Type IIb | 0.008(0.006)a | 0.017(0.010)a | 0.038(0.015)a | 0.019(0.003)a |
| Type I | *Methylobacter* | 0.001(0.001)a | 0.003(0.003)a | 0.030(0.024)a | 0.015(0.006)a |
|  | Unclassified Type Id | 0.000(0.000)a | 0.000(0.000)a | 0.000(0.000)a | 0.000(0.000)a |
|  | *Methylosarcina* | 0.000(0.000)a | 0.065(0.064)a | 0.000(0.000)a | 0.001(0.001)a |
|  | Unclassified Type Ib | 0.012(0.009)a | 0.006(0.004)a | 0.003(0.002)a | 0.003(0.003)a |
|  | *Methylomonas* | 0.002(0.002)a | 0.031(0.027)a | 0.009(0.008)a | 0.002(0.002)a |
|  | *Methylomicrobium* | 0.000(0.000)a | 0.000(0.000)a | 0.003(0.003)a | 0.000(0.000)a |
|  | Unclassified Type Ia | 0.000(0.000)a | 0.015(0.015)a | 0.000(0.000)a | 0.000(0.000)a |
| Unclassified | Unclassified MOB | 0.030(0.011)a | 0.097(0.040)a | 0.144(0.044)a | 0.143(0.041)a |
| All Type II |  | 0.946(0.021)a | 0.746(0.073)ab | 0.723(0.058)b | 0.801(0.044)ab |
| All Type I |  | 0.050(0.019)b | 0.243(0.069)ab | 0.267(0.060)a | 0.197(0.044)ab |
| class | genera | LQa | LQb | LQc | LQd |
| Type II | *Methylocystis* | 0.636(0.061)a | 0.547(0.073)a | 0.521(0.088)a | 0.439(0.060)a |
|  | Unclassified Type IIb | 0.000(0.000)a | 0.000(0.000)a | 0.000(0.000)a | 0.000(0.000)a |
| Type I | *Methylobacter* | 0.074(0.038)a | 0.023(0.012)a | 0.061(0.024)a | 0.058(0.018)a |
|  | Unclassified Type Id | 0.118(0.043)a | 0.266(0.093)a | 0.324(0.086)a | 0.284(0.100)a |
|  | *Methylosarcina* | 0.018(0.017)a | 0.011(0.007)a | 0.016(0.009)a | 0.021(0.015)a |
|  | Unclassified Type Ib | 0.122(0.032)a | 0.102(0.027)ab | 0.029(0.016)b | 0.024(0.013)b |
|  | *Methylomonas* | 0.015(0.008)a | 0.042(0.027)a | 0.009(0.008)a | 0.059(0.038)a |
|  | *Methylomicrobium* | 0.005(0.005)b | 0.000(0.000)b | 0.005(0.003)b | 0.056(0.014)a |
|  | Unclassified Type Ia | 0.003(0.002)a | 0.000(0.000)a | 0.002(0.001)a | 0.002(0.002)a |
| Unclassified | Unclassified MOB | 0.008(0.004)a | 0.006(0.004)a | 0.033(0.015)a | 0.050(0.019)a |
| All Type II |  | 0.618(0.060)a | 0.529(0.075)a | 0.514(0.087)a | 0.426(0.057)a |
| All Type I |  | 0.382(0.059)a | 0.470(0.074)a | 0.485(0.087)a | 0.567(0.059)a |
| class | genera | ZYa | ZYb | ZYc | ZYd |
| Type II | *Methylocystis* | 0.349(0.079)a | 0.340(0.071)a | 0.306(0.062)a | 0.337(0.100)a |
|  | Unclassified Type IIb | 0.000(0.000)a | 0.000(0.000)a | 0.000(0.000)a | 0.000(0.000)a |
| Type I | *Methylobacter* | 0.058(0.017)a | 0.264(0.138)a | 0.112(0.036)a | 0.116(0.055)a |
|  | Unclassified Type Id | 0.149(0.095)a | 0.012(0.009)a | 0.106(0.069)a | 0.007(0.006)a |
|  | *Methylosarcina* | 0.187(0.048)a | 0.068(0.021)a | 0.074(0.029)a | 0.117(0.050)a |
|  | Unclassified Type Ib | 0.116(0.031)a | 0.106(0.029)a | 0.189(0.105)a | 0.106(0.034)a |
|  | *Methylomonas* | 0.077(0.042)a | 0.095(0.063)a | 0.072(0.023)a | 0.242(0.094)a |
|  | *Methylomicrobium* | 0.001(0.000)a | 0.002(0.001)a | 0.003(0.002)a | 0.012(0.011)a |
|  | Unclassified Type Ia | 0.010(0.003)a | 0.006(0.003)a | 0.029(0.017)a | 0.011(0.006)a |
| Unclassified | Unclassified MOB | 0.023(0.008)a | 0.065(0.035)a | 0.105(0.058)a | 0.037(0.019)a |
| All Type II |  | 0.337(0.080)a | 0.335(0.071)a | 0.302(0.059)a | 0.321(0.095)a |
| All Type I |  | 0.631(0.089)a | 0.623(0.083)a | 0.694(0.059)a | 0.662(0.097)a |
| class | genera | SGHa | SGHb | SGHc | SGHd |
| Type II | *Methylocystis* | 0.207(0.206)a | 0.000(0.000)a | 0.000(0.000)a | 0.052(0.026)a |
|  | Unclassified Type IIb | 0.000(0.000)a | 0.000(0.000)a | 0.000(0.000)a | 0.000(0.000)a |
| Type I | *Methylobacter* | 0.188(0.098)a | 0.132(0.078)a | 0.510(0.251)a | 0.459(0.213)a |
|  | Unclassified Type Id | 0.000(0.000)a | 0.000(0.000)a | 0.001(0.001)a | 0.000(0.000)a |
|  | *Methylosarcina* | 0.509(0.181)a | 0.558(0.231)a | 0.341(0.194)a | 0.397(0.227)a |
|  | Unclassified Type Ib | 0.056(0.022)a | 0.067(0.021)a | 0.133(0.061)a | 0.069(0.045)a |
|  | *Methylomonas* | 0.029(0.029)a | 0.239(0.239)a | 0.014(0.014)a | 0.023(0.021)a |
|  | *Methylomicrobium* | 0.002(0.002)a | 0.002(0.002)a | 0.002(0.002)a | 0.000(0.000)a |
|  | Unclassified Type Ia | 0.000(0.000)a | 0.000(0.000)a | 0.000(0.000)a | 0.000(0.000)a |
| Unclassified | Unclassified MOB | 0.000(0.000)a | 0.001(0.001)a | 0.000(0.000)a | 0.000(0.000)a |
| All Type II |  | 0.169(0.167)a | 0.000(0.000)a | 0.000(0.000)a | 0.047(0.021)a |
| All Type I |  | 0.825(0.173)a | 1.000(0.000)a | 1.000(0.000)a | 0.953(0.021)a |
| class | genera | MQ | LQ | ZY | SGH |
| Type II | *Methylocystis* | 0.785(0.029)a | 0.522(0.036)b | 0.323(0.037)c | 0.062(0.051)d |
|  | Unclassified Type IIb | 0.019(0.004)a | 0.000(0.000)b | 0.000(0.000)b | 0.000(0.000)b |
| Type I | *Methylobacter* | 0.013(0.007)c | 0.053(0.012)bc | 0.131(0.035)b | 0.291(0.084)a |
|  | Unclassified Type Id | 0.000(0.000)b | 0.242(0.041)a | 0.047(0.020)b | 0.000(0.000)b |
|  | *Methylosarcina* | 0.016(0.015)b | 0.016(0.006)b | 0.098(0.018)b | 0.409(0.086)a |
|  | Unclassified Type Ib | 0.003(0.002)c | 0.068(0.014)b | 0.130(0.029)a | 0.071(0.017)abc |
|  | *Methylomonas* | 0.010(0.006)b | 0.032(0.012)b | 0.119(0.032)a | 0.063(0.046)ab |
|  | *Methylomicrobium* | 0.001(0.001)b | 0.016(0.005)a | 0.004(0.002)b | 0.001(0.001)ab |
|  | Unclassified Type Ia | 0.003(0.003)ab | 0.003(0.001)b | 0.014(0.004)a | 0.000(0.000)ab |
| Unclassified | Unclassified MOB | 0.142(0.023)a | 0.047(0.008)b | 0.111(0.031)ab | 0.099(0.024)ab |
| All Type II |  | 0.804(0.029)a | 0.522(0.036)b | 0.323(0.037)c | 0.063(0.051)d |
| All Type I |  | 0.189(0.029)d | 0.476(0.036)c | 0.654(0.040)b | 0.935(0.053)a |

**Table S7.** ﻿Comparison of PMORs and *pmoA* gene abundance. For abbreviations, see Table S2.

|  | MQa | MQb | MQc | MQd |
| --- | --- | --- | --- | --- |
| log pmoA gene copies (g^-1^dry soil) | 7.663(0.834)a | 7.061(0.532)a | 6.413(0.489)a | 7.363(0.366)a |
| PMORs [ng g^-1^(dry soil) day^-1^] | 661.935(31.944)a | 661.935(31.944)a | 695.080(28.550)a | 743.565(63.611)a |
|  | LQa | LQb | LQc | LQd |
| log pmoA gene copies (g^-1^dry soil) | 7.598(0.267)a | 7.327(0.254)a | 7.579(0.138)a | 6.954(0.182)a |
| PMORs [ng g^-1^(dry soil) day^-1^] | 727.745(14.751)a | 667.825(26.202)a | 678.147(23.392)a | 659.825(40.211)a |
|  | ZYa | ZYb | ZYc | ZYd |
| log pmoA gene copies (g^-1^dry soil) | 6.614(0.290)a | 6.938(0.564)a | 7.444(0.224)a | 6.565(0.459)a |
| PMORs [ng g^-1^(dry soil) day^-1^] | 543.040(42.076)a | 541.254(17.635)a | 425.825(47.470)a | 439.946(44.984)a |
|  | SGHa | SGHb | SGHc | SGHd |
| log pmoA gene copies (g^-1^dry soil) | 6.687(0.361)a | 6.854(0.191)a | 4.402(1.817)a | 4.850(1.804)a |
| PMORs [ng g^-1^(dry soil) day^-1^] | 252.177(25.727)a | 155.160(14.340)a | 134.404(40.239)a | 165.459(58.302)a |
|  | MQ | LQ | ZY | SGH |
| log pmoA gene copies (g^-1^dry soil) | 7.13(0.29)a | 7.36(0.11)a | 6.89(0.20)ab | 5.77(0.60)b |
| PMORs [ng g^-1^(dry soil) day^-1^] | 681.91(22.33)a | 683.39(13.99)a | 485.53(22.16)b | 182.60(20.99)c |

**Table S8.** The nodes identified as connectors / Module-hubs of MOB networks in four sites. For abbreviations, see Table S2.

| Site | ASV ID | Role | Relative abundance (%) | Genus | Class |
| --- | --- | --- | --- | --- | --- |
| LQ | ASV195 | Module_hubs | 0.065 | *Methylobacter* | Type I |
|  | ASV552 | Module_hubs | 2.959 | *Methylocystis* | Type II |
|  | ASV450 | Module_hubs | 1.936 | *Methylobacter* | Type I |
|  | ASV544 | Connector | 1.473 | Unclassified Type Id | Type I |
|  | ASV645 | Connector | 0.824 | *Methylobacter* | Type I |
| ZY | ASV980 | Module_hubs | 0.098 | *Methylosarcina* | Type I |
|  | ASV984 | Module_hubs | 0.121 | Unclassified Type Ib | Type I |
|  | ASV138 | Module_hubs | 0.038 | Unclassified Type Ia | Type I |
|  | ASV1222 | Module_hubs | 1.290 | *Methylocystis* | Type II |
|  | ASV1271 | Module_hubs | 0.038 | Unclassified Type Id | Type I |
|  | ASV262 | Module_hubs | 0.012 | *Methylobacter* | Type I |
|  | ASV923 | Module_hubs | 0.009 | *Methylomonas* | Type I |
|  | ASV420 | Module_hubs | 0.015 | *Methylocystis* | Type II |
|  | ASV282 | Module_hubs | 0.015 | Unclassified MOB | Type I |
|  | ASV936 | Module_hubs | 0.015 | *Methylobacter* | Type I |
|  | ASV559 | Module_hubs | 0.018 | Unclassified Type Ib | Type I |
|  | ASV913 | Module_hubs | 1.207 | *Methylobacter* | Type I |
|  | ASV387 | Connector | 0.033 | *Methylocystis* | Type II |
|  | ASV573 | Connector | 0.015 | *Methylobacter* | Type I |
|  | ASV526 | Connector | 0.027 | *Methylomonas* | Type I |
|  | ASV147 | Connector | 0.101 | *Methylobacter* | Type I |
|  | ASV458 | Connector | 0.071 | *Methylocystis* | Type II |
|  | ASV818 | Connector | 0.056 | Unclassified Type Ia | Type I |
|  | ASV1173 | Connector | 0.038 | *Methylomonas* | Type I |
|  | ASV375 | Connector | 0.745 | Unclassified Type Id | Type I |
|  | ASV793 | Connector | 0.139 | Unclassified Type Ib | Type I |
| SGH | ASV1136 | Module_hubs | 0.037 | *Methylobacter* | Type I |


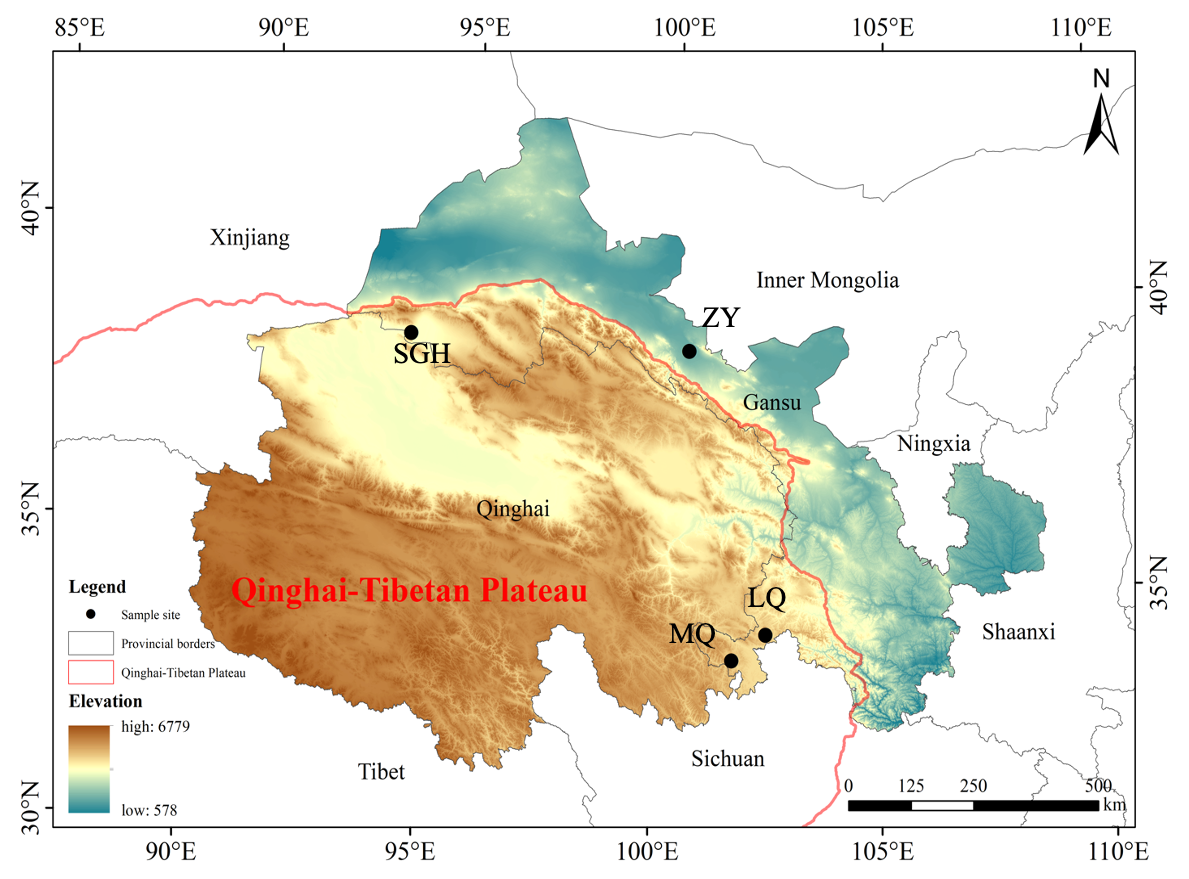


**Fig S1.** Research sites on the edge of northeastern Qinghai-Tibet Plateau. The data is provided by STRMdem (<https://earthexplorer.usgs.gov/>). This figure is identical to that published in our previous study (He *et al*., 2025). For abbreviations, see Table S2.


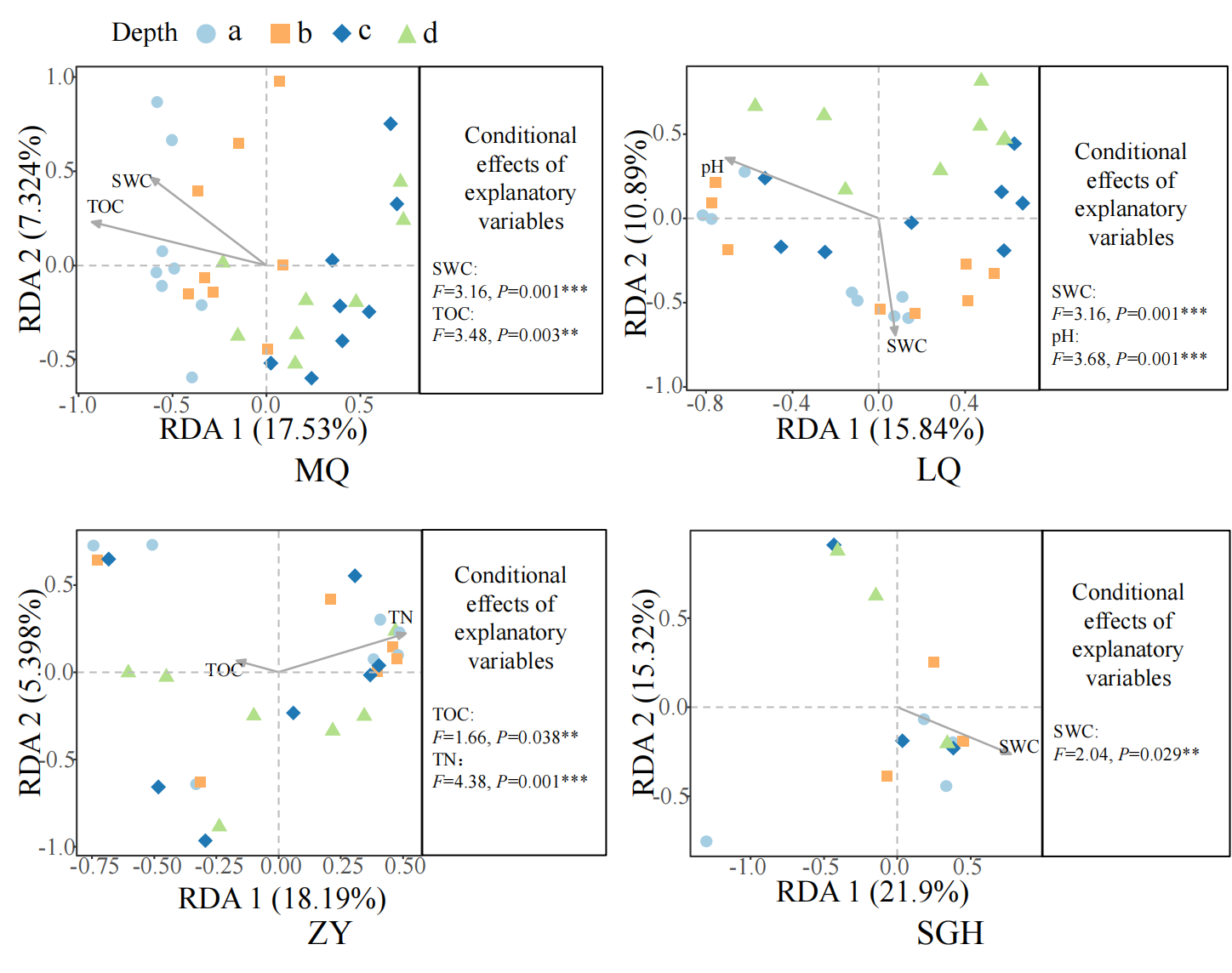


**Fig. S2** Community composition of MOB across soil depths at each site and their potential drivers. Shown are RDA ordination biplots with only significant explanatory variables (*P* < 0.05, conditional effects). For abbreviations, see Table S2.


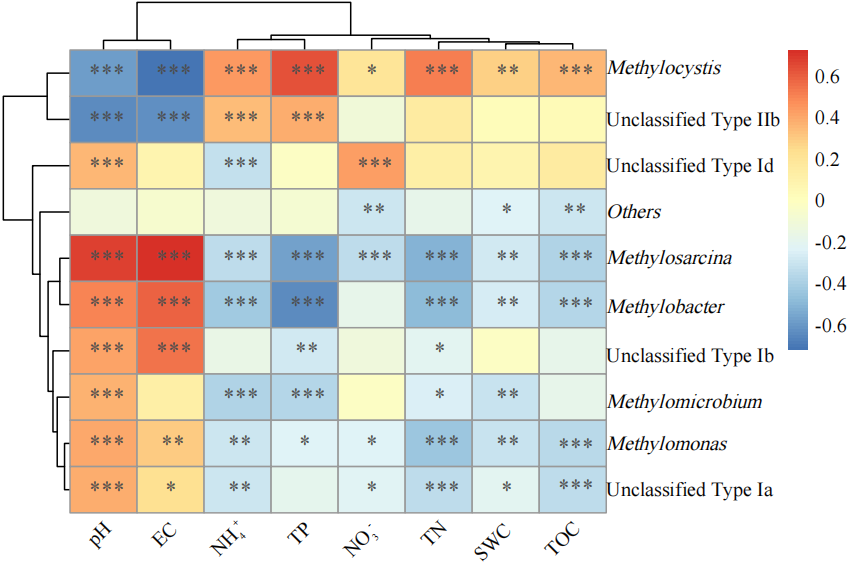


**Fig. S3** Heatmap analysis between the environmental factors and the top 10 genus relative abundance based on Wilcoxon Distance. ****P*<0.001; ** *P*<0.01; * *P*<0.05. For abbreviations, see Table S2.

**Fig. S4.** Network topological features of degree distribution patterns in four habitats. For abbreviations, see Table S2.

**Fig. S5.** The average degree and natural connectivity of microbial network within four sites. For abbreviations, see Table S2.


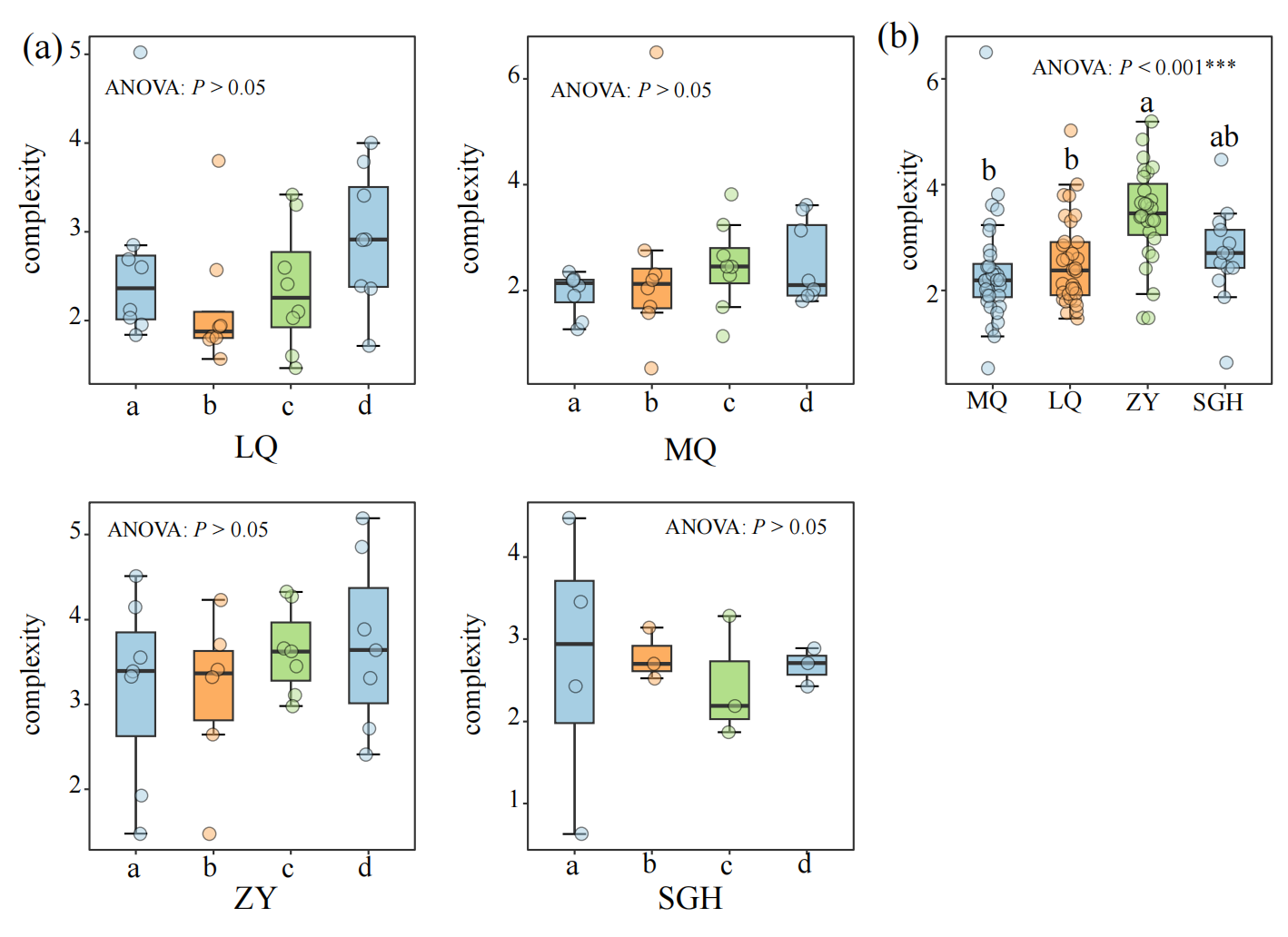


**Fig. S6.** Comparison of microbial subnetwork complexity across soil depths at four sites (a) and among sites (b). In (a), different letters denote soil depths from top to bottom. In (b), different letters indicate significant differences (*P* < 0.05) according to Tukey’s honest significant difference (HSD) test. Group differences were evaluated using one-way ANOVA. For abbreviations, see Table S2.


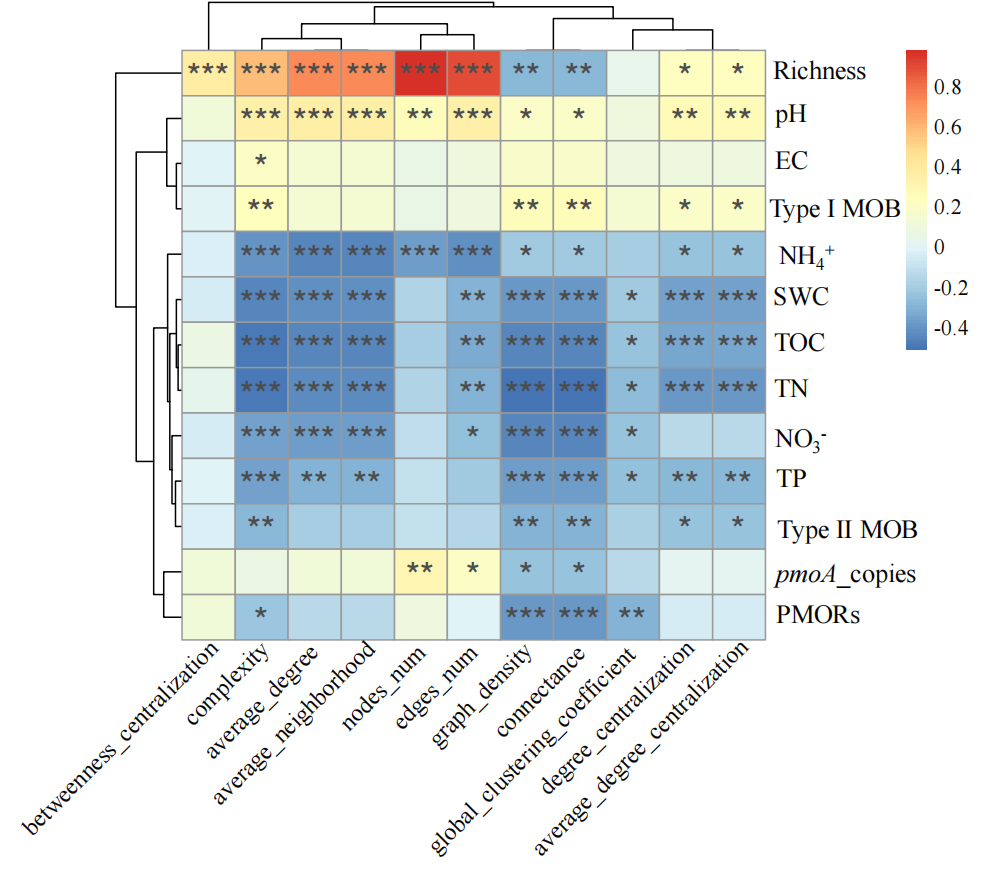


**Fig. S7** Heatmap analysis between the soil properties, MOB richness, type I/II MOB relative abundance, *pmoA* copies and the subnetwork topological properties based on Wilcoxon Distance. ***p<0.001; ** p<0.01; * p<0.05. For abbreviations, see Table S2.

Reference:

He, K., Zhao, J., Pan, J., Zhang, Q., and Feng, H. (2025) Coexistence networks of soil methanogens are closely tied to methane generation in wetlands on the northeastern of the Qinghai–Tibet Plateau. *Front Microbiol* **16**: 1616051.
